# Distinct Ligand‐ and Metal‐Centered Phosphorescence in a Terbium Carbazolyl Complex

**DOI:** 10.1002/chem.202503208

**Published:** 2025-12-02

**Authors:** Xiaofei Sun, Frederic Krätschmer, Julia Feye, Marcel Lukanowski, Dominik Graf, Sergei Lebedkin, Manfred Kappes, Florian Weigend, Peter W. Roesky, Alexander Hinz

**Affiliations:** ^1^ Karlsruhe Institute of Technology (KIT) Institute of Inorganic Chemistry (AOC) Karlsruhe Germany; ^2^ Karlsruhe Institute of Technology (KIT) Institute For Quantum Materials and Technologies (IQMT) Karlsruhe Germany; ^3^ Karlsruhe Institute of Technology (KIT) Institute of Nanotechnology (INT) Eggenstein‐Leopoldshafen Germany

**Keywords:** bulky Ligand, carbazole, lanthanum, luminescence, terbium

## Abstract

Carbazolide complexes of lanthanum and terbium with cyclooctatetraenediide (COT) and THF coligands of the type [(^dtbp^Cbz)LnCOT(thf)_n_] (n = 2 for La, 1 for Tb) were synthesized by salt metathesis reactions. The THF molecules were found to be labile, and drying under vacuum led to their partial removal with concomitant formation of the dinuclear complexes [(^dtbp^Cbz)_2_Ln_2_(COT)_2_(thf)]. The luminescence of both lanthanum and terbium complexes was investigated, and at cryogenic temperatures, strongly temperature‐dependent phosphorescence was observed. The terbium complexes show the expected element‐characteristic emission with narrow lines between 480 and 700 nm upon excitation at 370 nm. Beyond that, broad emission was induced selectively by excitation at lower energy. Related phosphorescence was found for the lanthanum complex, which implies intra‐ or inter‐ligand excitation as source for the latter. This interpretation was corroborated by TD‐DFT computations.

## Graphical abstract

Terbium and lanthanum carbazolide complexes show two distinct modes of luminescence: Strongly temperature‐dependent narrow‐line terbium emission could be induced. In addition, both the terbium and the lanthanum complexes show broad‐line interligand emission processes with excited states of microsecond life‐time.

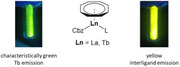

## 1 Introduction

**SCHEME 1:**
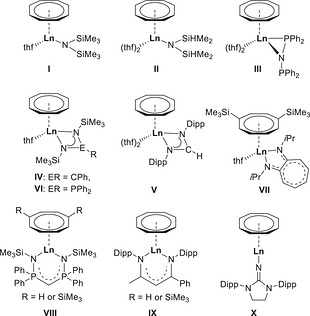
Selected examples of heteroleptic lanthanide cyclooctatetraenediide complexes with monoanionic N‐donors.

The aromatic, 10π‐electron cyclooctatetraenide (COT) derivatives serve as important building blocks in organolanthanide chemistry. The steric demand of the COT ligands can be tuned by introducing, for example, different silyl‐substituents at the 1‐ and 4‐position. During the past decades, different homoleptic structural motifs have been disclosed by using solely the COT derivatives. These range from simple double‐decker [1, 2] and triple‐decker [3] species to even larger multi‐decker compounds [4, 5, 6]. Apart from the homoleptic ones, a certain number of heteroleptic COT‐containing sandwich structures have also been isolated, most of them utilizing other π‐coordinating coligands [7, 8, 9, 10, 11]. Introduced in place of cyclic carbon‐based ligands, anionic N‐donor ligands have expanded the scope of heteroleptic Ln‐COT compounds as suitable anchor ligands for the hard trivalent lanthanides. Notable examples include amides (Scheme 1, **I**‐**III**) [12, 13, 14], amidinates (**IV**, **V**) [15, 16], iminophosphonamides (**V** **I**) [15], aminotroponiminates (**VII**) [17], bis(phosphinimino)methanides (**VIII**) [18, 19, 20], β‐diketiminates (**IX**) [21], and imido ligands (**X**) [22, 23] as coligands (Scheme 1). In addition to their unique structural motifs, some of those compounds have also been investigated in terms of their catalytic behavior. For example, the samarium complex [{CH(PPh_2_NSiMe_3_)_2_}Sm(η^8^‐C_8_H_8_)] was found to be an efficient catalyst for intramolecular hydroamination/cyclisation reaction of terminal aminoolefins and alkynes [20]. While the catalytic aspects of such compounds have garnered attention, their optical properties remain relatively unexplored.

Carbazole derivatives represent another group of promising N‐donor ligands for heteroleptic Ln‐COT compounds. Recent research on carbazoles has put a primary focus on their optical properties, with a purpose of their use in organic light‐emitting diodes and field‐effect transistors (OLED and OFET). Variation in substitution patterns on the carbazole scaffold and coordination to diverse metals, have unveiled intriguing luminescent properties.

In coordination chemistry, various monodentate and tridentate pincer‐type carbazolides have been utilized as ligands for various metal ions. In previous work, our group prepared a series of alkali‐metal carbazolides bearing the sterically demanding carbazolyl substituent ^dtbp^Cbz (^dtbp^Cbz = 1,8‐bis(3,5‐di‐*tert*‐butyl‐phenyl)‐3,6‐di‐*tert*‐butyl‐carbazolyl), which show efficient fluorescence with the emission maxima across a broad spectral range [24].

The aim of this work is an introduction of the ^dtbp^Cbz ligand into heteroleptic Ln‐COT compounds and characterization of its “antenna” effect for sensitization of trivalent lanthanides for which element‐characteristic narrowband luminescence is expected. In this contribution, we report on the synthesis and structures of the lanthanum and terbium carbazolide‐COT complexes and their photophysical properties.

## 2 Results and Discussion

### 2.1 Syntheses and Structures

**SCHEME 2:**
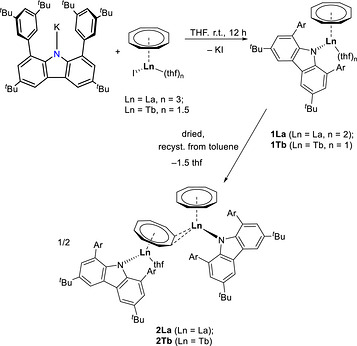
Synthesis of the La and Tb carbazolide COT complexes.

When potassium carbazolide was treated with [Tb(COT)I(thf)_1.5_] [25] in THF (Scheme 2), the initially yellow solution immediately darkened. After stirring overnight and evaporation of all volatiles at ambient temperature, the greenish residue was extracted with toluene and filtered off. From the toluene solution, crystalline material was obtained after concentration. After discarding the supernatant and washing the crystals with *n*‐hexane, **1** **Tb** was obtained in 47% yield. To prepare **2** **Tb**, crystals of **1** **Tb** were ground with a spatula and further dried for 60 min at ambient temperature. Recrystallisation of the material from toluene afforded **2** **Tb** in 81% yield. The lanthanum analogues were prepared for comparison. Similarly, the reaction between potassium carbazolide [(^dtbp^Cbz)K] [26] and [La(COT)I(thf)_3_] [27, 28] was carried out in THF. After stirring the reaction mixture at room temperature for 12 h, the insoluble materials (KI) were filtered off. Layering the concentrated THF solution with *n*‐pentane led to the formation of yellow crystals in 44% yield. The isolated single crystals of complex **1La** were dried under vacuum and redissolved in C_6_D_6_ for NMR spectroscopy. In the ^1^H NMR spectrum, two singlet signals were detected at 1.59 and 1.42 ppm in 2:1 molar ratio for the two types of *t*Bu groups. The ring proton signals for the COT were found at 5.58 ppm. Out drying procedure removed effectively one and a half THF molecules from the complex. Recrystallization from toluene at room temperature led to the formation of complex **2La** in 72% crystalline yield. It should be noted that neither **1** nor **2** can be dried at elevated temperatures without decomposition, which yields ^dtbp^CbzH and insoluble yellowish material.

**FIGURE 1:**
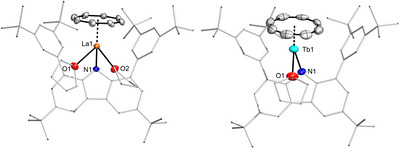
Molecular structures of **1La** and **1** **Tb**, thermal ellipsoids at 50% probability at 130 and 180 K, respectively. Selected bond lengths [Å] and angles [°] for **1La**: N1–La1 2.579(3), O1–La1 2.627(2), N1–La1–O1 90.39(8), La1–NC4 plane 37.0°, 2.061 Å; for **1Tb**: N1–Tb1 2.353(3), O1–Tb1 2.418(2), N1–Tb1–O1 81.24(9), Tb1–NC4 plane 35.8°, 1.914 Å.

Complex **1Tb** was crystallized from toluene as a THF solvate in the space group *P*2_1_/*n* (Figure 1). The COT ligand was modelled disordered over two positions. The complex can be described as two‐legged ‘piano stool’ motif. Key interatomic distances are N–Tb 2.353(3) Å and O–Tb 2.418(2) Å with an N–Tb–O angle of 81.24(9)°. The Tb–COT interaction is characterized by a Tb– COT centroid distance of 1.788 Å, while the average Tb–C distance is 2.568 Å. The Tb atom is 1.914 Å away from the central NC_4_ plane of the carbazole, with an angle of the N–Tb vector to that plane of 35.8°.

Complex **1La** crystallized from a THF/*n*‐pentane layered solvent mixture in the orthorhombic space group *Pnma* and the asymmetric unit cell contains half of the molecule, with the La–N bond located on a mirror plane (Figure 1). The molecular structure of **1La** closely resembles that of **Tb**, however, due to the larger ionic radius of La^3+^ compared to Tb^3+^, two THF molecules coordinate to the La atom. The planar cyclooctatetraenide ring is η^8^‐bound with a slight variation of the La–C distances from 2.691(6) to 2.725(7) Å, resulting in a three‐legged ‘piano stool’ geometry.

The key metrics are N–La 2.579(3) Å, O–La 2.627(2) Å with N‐La‐O 90.39(8)° and O‐La‐O 69.54(11)°. The La–COT centroid and average La─C distances are 2.026 Å and 2.729 Å, respectively. The La─N bond is bent out of the NC_4_ plane by 37.0° resulting in a distance of 2.061 Å between La and that plane. We note that the above N─La distance is significantly longer than in the comparable La‐amide complex [(COT)La[N(SiHMe_2_)_2_](thf)_2_] (2.410(2) Å) [13].

**FIGURE 2:**
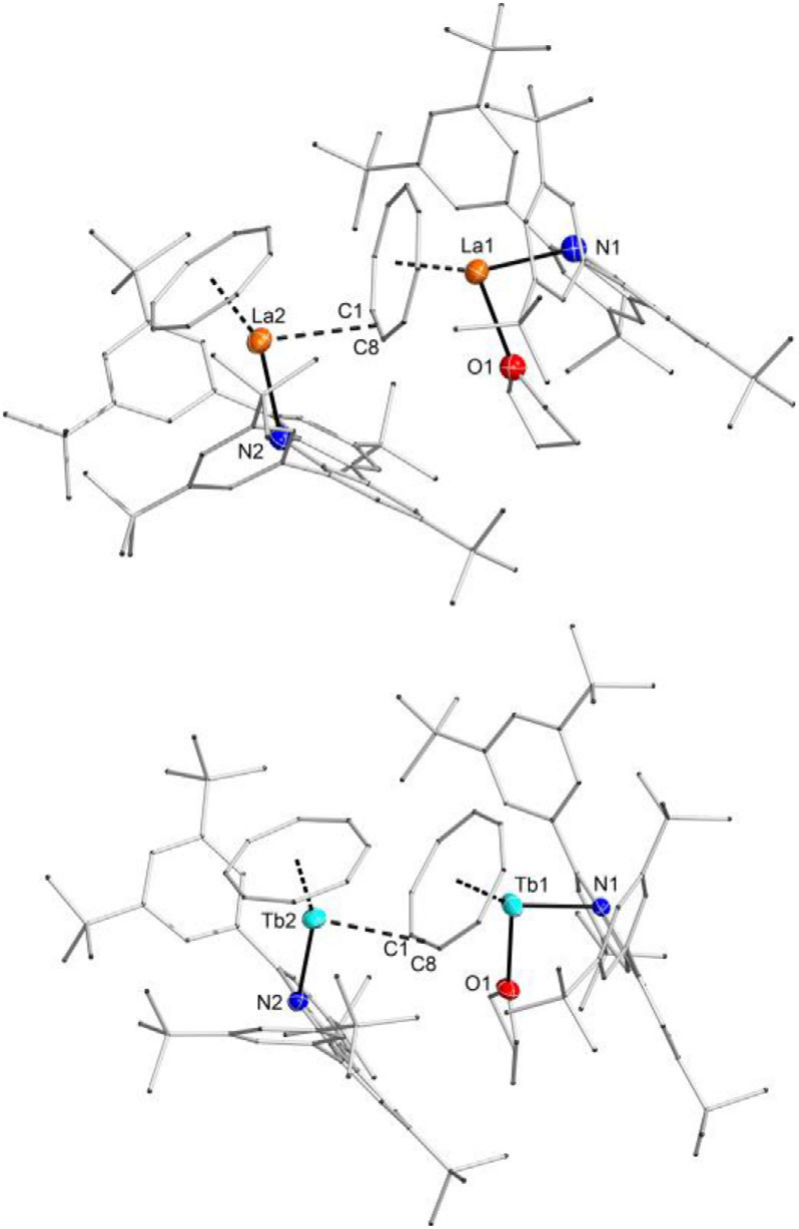
Structures of **2La** and **2** **Tb**. Thermal ellipsoids at 50% probability at 180 and 100 K, respectively. Selected bond lengths [Å] and angles [°] for **2Tb**: N–Tb1 2.326(2), O–Tb1 2.372(2), N–Tb1–O 82.01(8), Tb1–NC4 plane –34.4°, 1.921 Å, N–Tb2 2.357(2), C–Tb2 2.93(2), N–Tb2–C 77.2(2); for **2La**: N–La1 2.440(2), O–La1 2.530(1), N–La1–O 78.7(4), La1–NC4 plane –40.9°, 1.833 Å, N–La2 2.507(14), C–La2 2.98(2), N–La2–C 75.9(6), La2–NC4 plane –41.2°, 1.896 Å.

Upon removal of coordinated THF molecules by drying **1Tb** and **1La** and recrystallisation from toluene, complexes **2** **Tb** and **2La** were obtained (Figure 2). In their structures, one moiety is very similar to **1** **Tb** and **1La**. The other one features a related coordination sphere, where the coordinated THF is removed and replaced by a long η^1^‐contact between C1 and the metal atom Tb2 and La2, respectively. The Ln2–COT centroid contacts are shorter than their Ln1 analogues, as are the average Ln–C contacts (**2Tb**: Tb1–COT centroid 1.810 Å, average Tb1–C 2.583 Å; Tb2–COT centroid major component 1.776 Å, average Tb2–C 2.554 Å; **2La**: La1–COT centroid 2.009 Å, La1–C average 2.717 Å; La2–COT centroid major component 1.973 Å, La2–C average 2.693 Å).

Tb2 displays the smallest angle between the Tb2–N vector and the NC_4_ plane (44.2°) and thus the smallest deviation of Tb2 from that plane (1.697 Å), placing the atom in the “pocket” provided by the Cbz ligand and potentially enabling π interactions.

### 2.2 Photoluminescence Properties

**FIGURE 3:**
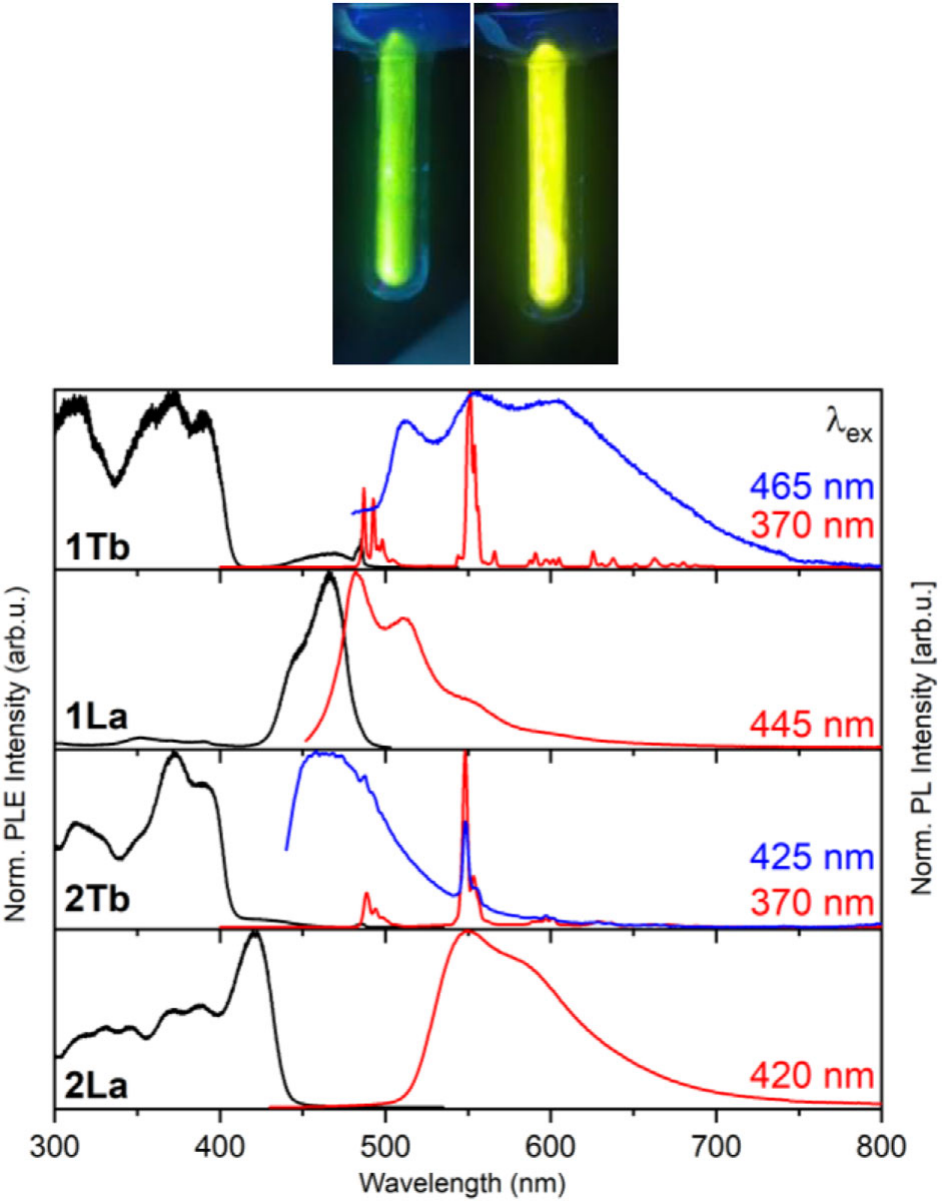
Top: Photograph of emission of **1** **Tb** (left) and **1La** (right) in frozen THF solution upon excitation with a (254/366 nm) UV lamp. Bottom: Normalized PLE (black) and PL (red and blue, excitation values given) spectra of **1** **Tb**, **1La**, **2** **Tb** and **2La** at 77K. Complexes **1** **Tb** and **1La** were measured in frozen THF solution, **2Tb** and **2La** as neat solids.

As could be expected, photoluminescence (PL) of **1La** and **2La** shares some features with the PL of the alkali‐metal complexes possessing the same ^dtbp^Cbz ligand [24]. Indeed, the lanthanum ion does not show element‐specific luminescence, hence the PL spectra observed for **1La** and **2La** are assumed to similarly originate from the negatively charged carbazolyl scaffold. Those features include the PL excitation (PLE) onset at ca. 450–500 nm and broad emission centered at ca. 500–550 nm (Figure 3). However, in contrast to the fluorescence of the alkali‐metal complexes [24], **1La** and **2La** only emit phosphorescence as indicated by microsecond‐long decay times (**1** **La**: τ = 6 µs at 77 K, Figure S13). Accordingly, the La ion appears to provide for very efficient intersystem conversion (ISC) to a triplet excited state in the ^dtbp^Cbz ligand. With the exception of **2La**, phosphorescence demonstrates a relatively small Stokes shift and therefore is spectrally close to the above fluorescence. In contrast to the latter [24], it is bright at cryogenic temperatures, but decreases strongly above ∼150 K. This observation has been made both for fast‐frozen THF solutions of **1La** at 77 K (containing monomeric species) and solid samples of **2La** (where crystallographically authenticated dimeric species cause the emission). At room temperature, the La complexes are practically nonluminescent. Figure S9 illustrates the PL temperature dependence for solid **2La**. In case of the terbium complexes **1Tb** and **2** **Tb** (PL studied in THF solution and in solid (polycrystalline) state, respectively), a bright emission typical for Tb(III) (perceived as a green light) is observed at low temperatures. For both **1Tb** and **2** **Tb,** excitation with longer wavelengths also led to broad carbazolyl emission (Figure 3, blue lines). The Tb emission consists of the characteristic structured bands corresponding to the major ^5^D_4_→^7^F_6_, ^5^D_4_→^7^F_5_ transitions (at ca. 485 and 550 nm) as well as the low intensity bands of the ^5^D_4_→^7^F_4_ and ^5^D_4_→^7^F_3_ transitions (at ca. 595 and 630 nm). The lifetime of the excited state at 77 K amounts to 110 µs (average value from a biexponential fit) for solid **2** **Tb** and 380 µs for **1** **Tb** dissolved in THF. Typically, luminescent Tb(III) complexes show only a moderate temperature dependence of the PL properties and many of them emit efficiently even in aqueous solutions [29]. The temperature dependence of the Tb(III) emission in **1** and **2** **Tb** follows, however, that of the carbazolyl phosphorescence in **1La** and **2La**: it is bright below ∼100 K, but strongly decreases above ∼150 K (Figure S10). At room temperature, Tb(III) emission was practically nonobservable in dissolved **1Tb**, in solid **2Tb** it was estimated to drop by a factor of 100. A corresponding quantum efficiency was estimated as 0.3% (by using an integrating sphere and excitation at 350 nm). The PL efficiency thus approaches a ∼30% level below 50 K, in accordance with the bright green emission of **2Tb** visually observed at low temperatures. On the other hand, the emission decay (tens to few hundreds of microseconds) only moderately depends on the temperature (Figure S14), indicating that the intensity decrease is due to a breakdown in energy transfer to the Tb(III) ion. A similar scenario likely also occurs in **1Tb**. The mechanistic details of such thermally induced decrease in energy transfer to Tb(III) are not clear at present. One can note here that thermal quenching of Tb(III) emission has been commonly observed in inorganic materials, albeit relatively rarely at low temperatures [30]. However, this quenching is due to thermally activated electron transfer from the Tb(III) ^5^D_4_ level to the conduction band of a specific inorganic host and thus different from that in **1Tb** and **2** **Tb**.

The emission peaks of **1** **Tb** and **2** **Tb** shown in Figure 3 have a bandwidth of ca. 0.5 nm defined by instrumental resolution. In order to determine the intrinsic bandwidth, the emission of polycrystalline **2Tb** was also measured at 3.2 K using a long focal length spectrograph with a resolution of ca. 0.015 nm (for details see the Supporting Information). Figure S11 shows a high‐resolution spectrum of the ^5^D_4_→^7^F_5_ transition between 545–550 nm, indicating an intrinsic FWHM value for the major multiplet peak at 547.5 nm as ca. 0.1 nm (3 cm^−1^). Such sharp emission suggests a high degree of structural order in the sample. The spectrum appears unexpectedly simple considering the presence of two Tb ions in the dimeric molecule of **2** **Tb**. However, their local surroundings are rather similar (Figure 2) and it is likely that their emissions overlap within the above bandwidth.

### 2.3 Computational Studies

Time‐dependent density functional theory calculations [31, 32] at the scalar‐relativistic X2C/PBE0 level using x2c/TZVPall‐2c basis sets [33, 34] have been done to gain further insight into the character of the lowest excitations. Singlet vertical excitations were calculated for the 40 energetically lowest excitations for both **1La** and **1** **Tb** in their monomeric crystal structures. The nonrelaxed overall density difference to the ground state was obtained and plotted as described previously [35].

**FIGURE 4:**
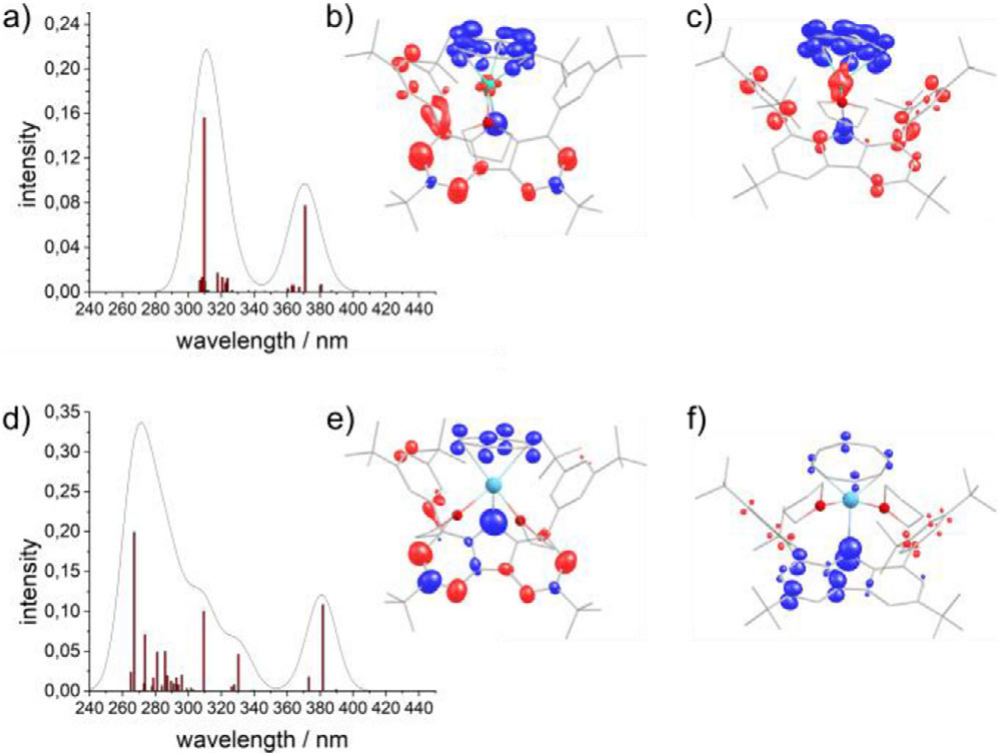
Calculated excitations and spectrum simulated by Gaussian broadening (half‐width 10 nm) of **1** **Tb** (a) and **1La** (d). Differences in electron densities of the first (b, e)/second (c, f) excitation band (wavelengths larger/smaller than 350 nm) and the ground state. Blue/red color indicates a decrease/increase of electron density upon excitation; contours are drawn at ±0.003 au.

For both compounds, the lowest 40 excitations were calculated and the two lowest bands that are clearly separated from the subsequent ones were analyzed, see Figure 4, left. The maxima of the first band at 382 nm for **1La** and 371 nm for **1** **Tb** are in reasonable agreement with those in the measured absorption spectra, 445 nm for **1La** and 370 nm for **1** **Tb**, see Figure 3, well reproducing the red‐shift for **1La** compared to **1Tb** (Figure 4a, d). For the latter, the nonrelaxed density difference (Figure 4b) indicates a transition of electron density (from blue to red) upon excitation mainly from the π‐system of the COT ring to the π‐systems of the phenyl rings and of the carbazole unit (except of the N position), but also minor contributions to the f shell of Tb are visible; according to a Mulliken population analysis [36], the f occupation is increased by 0.03 electrons for this band which is very low. Also, for **1La,** the lowest excitation band mainly is a ligand‐ligand excitation, but here the La(f) orbitals are not involved (Figure 4e).

In addition, for **1** **Tb** six very minor excitations in the low enery range from roughly 37000 to 1960 nm are found, see Figure S14. The most dominant excitation lies at 2570 nm (3890 cm^−1^ resp.) and suggests a very weak 4f–4f excitation of the single minority spin f electron. These transitions are allowed due to distortion of the atomic shell via the coordinating COT and carbazolide ligands. Changes within the orbitals of the f shell amount to 0.2 electrons. A detailed orbital‐resolved analysis of the transitions is given in the Supporting Information. The second excitation band for **1** **Tb** (Figure 4c) features an excitation of the π‐system of the COT ring and the N atom to the Tb atom (for individual orbital contributions, see Tables S2, S3) which is in line with the experimental results. Here, the electron density at Tb increases by a total of 0.13 electrons (0.05 for s, and 0.08 for f). This feature is clearly missing in the second excitation band for **1La** (Figure 4f), probably due to the higher energy of the f orbitals for La compared to Tb.

It is likely that the (small) participation of the f shell in the excitations present in **1** **Tb**, but not in **1La**, is preserved also for the emission. This leads to the specific PLE spectral lines for **1La** broad charge–transfer transitions involving the π‐systems of the different rings, while for **1** **Tb,** additional f‐f transitions at wavelengths greater than ∼2000 nm as well as a ligand–ligand transition for lower excitation wavelength were substantiated.

## 3 Conclusions

Salt metathesis with elimination of potassium iodide enabled the synthesis of lanthanide COT carbazolide complexes. The THF content is variable: Initially, monomeric complexes were obtained, which, upon drying and recrystallizing, yielded dinuclear compounds. The former were used for luminescence study in THF solution, while the latter investigated as crystalline solids. In each instance, phosphorescence was observed. For the terbium complexes, in dependence of the excitation wavelength, either the element‐characteristic narrow line emission or broad line intraligand emission could be induced. The lanthanum complexes only show the latter phenomenon. Based on TD‐DFT computations the relevant excitations were identified as π(COT)→π*(Cbz/arene) transitions.

## Conflicts of Interest

The authors declare no conflict of interest.

## Supporting information




**Supporting Information File 1**: The raw data Supporting information was deposited at RADAR4Chem (DOI 10.22000/krgdd0mhdsavzmrg). Deposition Number(s) https://www.ccdc.cam.ac.uk/services/structures?id = 2476612 (for **1La**), 2476613 (for **1Tb**), 2476614 (for **2La**), 2476615 (for **2Tb**) contain the supplementary crystallographic data for this paper. These data are provided free of charge by the joint Cambridge Crystallographic Data Centre and Fachinformationszentrum Karlsruhe Access Structures service

**Supporting Information File 2**: chem70516‐sup‐0002‐SuppMat.zip

## Data Availability

The data that support the findings of this study are available in the supplementary material of this article.
