# Supplementary material for: Distinct Ligand‐ and Metal‐Centered Phosphorescence in a Terbium Carbazolyl Complex
Source: Chemistry. 2025 Dec 2;32(29):e03208. doi: 10.1002/chem.202503208 (PMC13451547; doi:10.1002/chem.202503208)
Supplement: Supplementary file 1 [file CHEM-32-e03208-s002.pdf]

Supporting Information for:

Distinct ligand- and metal-centered phosphorescence in a terbium carbazolyl complex

Xiaofei Sun, Frederic Krätschmer, Julia Feye, Marcel Lukanowski, Dominik Graf, Sergei Lebedkin, Manfred Kappes, Florian Weigend, Peter W. Roesky, Alexander Hinz

Table of Contents

I. Synthesis and characterization 2
I.1 General procedures 2
I.2 Synthesis of complex **1La** and **2La** 3
I.3 Synthesis of complex **1Tb** and **2Tb** 4
II. NMR spectra 5
III. IR spectra 6
IV. X-ray crystallography 7
IV.1 General methods 7
IV.2 Summary of crystal data 9
V. Photoluminescence 13
V.1 General methods 13
V.2 PL spectra 14
VI. Quantum chemical calculations 18
VII. References 25

I. Synthesis and characterization

I.1 General procedures

All air- and moisture-sensitive manipulations were performed under dry N_2 or Ar atmosphere using standard Schlenk techniques or in an argon-filled MBraun glovebox, unless otherwise stated. *n*-pentane and toluene were dried using an MBraun solvent purification system (SPS-800) and degassed. THF was distilled under nitrogen from potassium benzophenone ketyl. C_6D_6 and THF-d_8 were dried over Na-K alloy and degassed by freeze-pump-thaw cycles. CbzK[1] and [LnCOTI(thf)$_n$] (Ln = La, Tb)[2] were prepared according to the literature procedures. All other chemicals were obtained from commercial sources and used without further purification.

Elemental analyses were carried out with an Elementar vario MICRO cube.

NMR spectra were recorded on Bruker spectrometers (Avance Neo 300 MHz, Avance Neo 400 MHz or Avance III 400 MHz). Chemical shifts are referenced internally using signals of the residual protio solvent (^{1}H) or the solvent (^{13}C{^{1}H}) and are reported relative to tetramethylsilane (^{1}H, ^{13}C{^{1}H}). The multiplicity of the signals is indicated as s = singlet, d = doublet, dd = doublet of doublets, t = triplet, q = quartet, m = multiplet and br = broad. Assignments were determined based on unambiguous chemical shifts, coupling patterns and ^{13}C-DEPT experiments or 2D correlations (^{1}H-^{1}H COSY, ^{1}H-^{13}C HMQC and ^{1}H-^{13}C HMBC).

Infrared (IR) spectra were recorded in the region 4000–400 cm^{-1} on a Bruker Tensor 37 FTIR spectrometer equipped with a room temperature DLaTGS detector, a diamond attenuated total reflection (ATR) unit and a nitrogen-flushed chamber. In terms of their intensity, the signals were classified into different categories (vs = very strong, s = strong, m = medium, w = weak, and sh = shoulder).

I.2 Synthesis of complex 1La and 2La

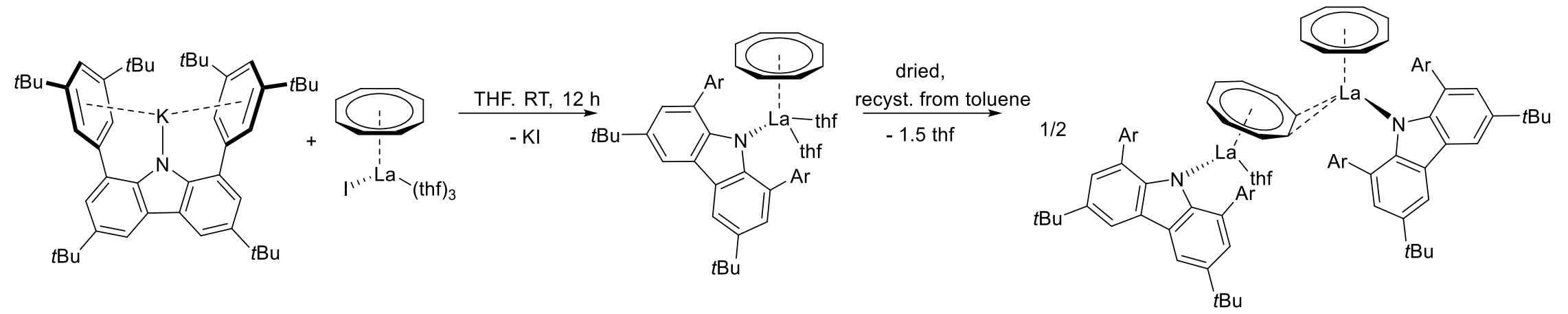

1La **2La**

1La: To a mixture of CbzK (100.0 mg, 0.144 mmol) and [La(COT)I(thf)$_3$] (84.5 mg, 0.144 mmol) was added 5 mL THF at room temperature. After stirring the reaction mixture for 12 h at room temperature, the solution was filtered to remove the metathesis salt KI. The remaining dark yellow solution was concentrated and layered with *n*-pentane to form complex **1La** as light-yellow crystals.

Single crystal X-ray diffraction analysis confirmed the identity of complex **1La**. For each La atom, there are two coordinated THF molecules. The crystals of **1La** were isolated and dried under vacuum for 1 h. According to NMR and elemental analysis, upon sufficient drying, only 0.5 coordinated THF molecule is left. Crystalline yield: 59 mg (0.061 mmol, 44%).

2La: The isolated crystals of complex **1La** were redissolved in toluene, and single crystals of complex **2La** could be obtained by slow evaporation of toluene at room temperature (41 mg, 0.022 mmol, 72%).

Anal. Calcd. For **2La**, $C_{56}H_{72}NLa$ 0.5(C_4H_8O) (934.12 g/mol): C 74.57; H 8.20; N 1.50. Found: C 73.77; H 7.72; N 1.79.

^{1}H NMR (400 MHz, THF-d_8): δ (ppm) = 7.95 (br s, 2H, CH_{Cbz}), 7.73 (br s, 2H, CH_{Cbz}), 7.63 (br s, 4H, CH_{Cbz}), 7.21-7.07 (m, 7H, 2H from CH_{Cbz} + 5H from CH_{tol}), 5.58 (s, 8H, CH_{COT}), 3.63-3.60 (*thf*), 1.79-1.76 (*thf*), 1.59 (s, 36H, CH_3), 1.42 (s, 18H, CH_3).

^{13}C{^{1}H} NMR (101 MHz, THF-d_8): δ (ppm) = 151.6 (C_q), 144.7 (C_q), 142.5 (C_q), 140.7 (C_q), 127.0 ($C^{2,7}$H), 126.2 (C_q), 125.5 (*o*-CH), 123.5 (*p*-CH), 115.6 ($C^{4,5}$H), 98.2 (CH$_{COT}$), 67.9 (CH$_{2\,thf}$), 36.0 (Ar-C(CH$_3$)$_3$), 35.2 (Cbz-C(CH$_3$)$_3$), 32.7 (Cbz-C(CH$_3$)$_3$), 32.6 (Ar-C(CH$_3$)$_3$), 25.8 (CH$_{2\,thf}$).

IR (ATR): $\tilde{\nu}$ (cm^{-1}) = 378 (vw), 386 (w), 395 (vw), 408 (vw), 420 (vw), 441 (vw), 464 (m), 502 (w), 519 (w), 532 (w), 549 (w), 591 (vw), 604 (vw), 621 (vw), 644 (m), 665 (w), 675 (w), 703 (vs), 727 (m), 744 (w), 751 (w), 771 (w), 781 (vw), 825 (w), 845 (m), 868 (s), 897 (w), 924 (w), 982 (m), 1021 (w), 1106 (w), 1151 (m), 1184 (m), 1202 (m), 1229 (vs), 1245 (s), 1264 (m), 1287 (m), 1340 (w), 1361 (s), 1379 (w), 1391 (m), 1436 (w), 1461 (m), 1476 (m), 1493 (w), 1589 (m), 2866 (m), 2905 (m), 2954 (s).

I.3 Synthesis of complex 1Tb and 2Tb

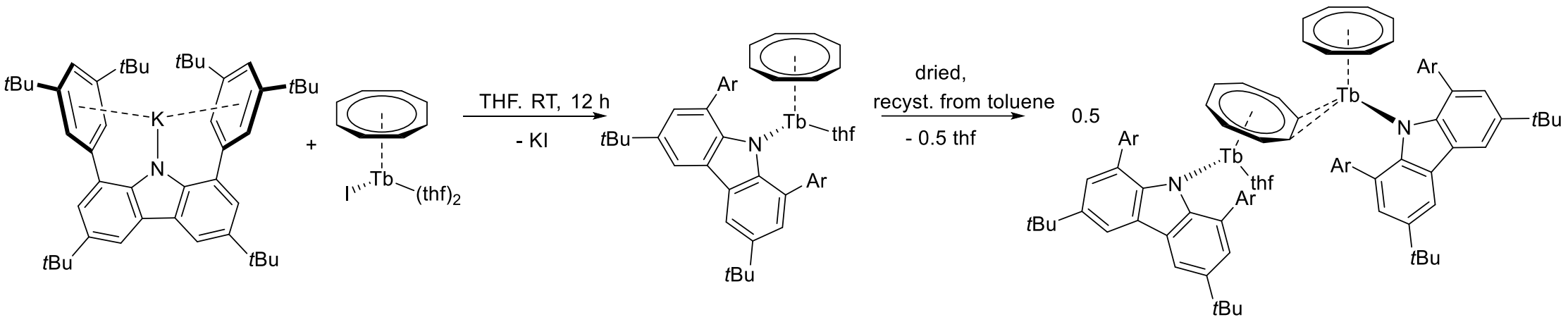

1Tb 2Tb

1Tb: To a mixture of CbzK (100.0 mg, 0.144 mmol) and Tb(COT)I(thf)$_2$ (77.0 mg, 0.144 mmol) was added 5 mL THF at room temperature. After stirring the reaction mixture for 12 h at room temperature, all volatiles were evaporated at room temperature. The yellowish residue solution was extracted with 5+1 ml of toluene, the filtered off toluene solution then concentrated to incipient crystallisation and left undisturbed concentrated to form complex **1Tb** as pale yellow crystals. Single crystal X-ray analysis confirmed the identity of complex **1Tb**. The supernatant was removed via syringe and the crystals were washed with 2x 0.5 ml of n-hexane. The crystals were then dried for 5 min under dynamic vacuum. Crystalline yield: 65 mg (0.066 mmol, 47%).

2Tb: The dried crystals of complex **1Tb** were ground to a fine powder and further dried in vaccum for 90 minutes. After redissolution in toluene and concentration of the solution, single crystals of complex **2Tb** could be obtained. Crystalline yield: 51 mg (0.053 mmol, 81%).

Anal. Calcd. For **2Tb**, $C_{56}H_{72}NTb$ 0.5(C_4H_8O) (954.18 g/mol): C 73.01; H 8.03; N 1.47. Found: C 73.39; H 7.98; N 1.90.

IR (ATR): $\tilde{\nu}$ (cm^{-1}) = 383 (w), 462 (w), 503 (w), 549 (w), 644 (w), 676 (w), 703 (vs), 771 (w), 845 (m), 868 (s), 897 (w), 927 (w), 981 (vw), 1021 (w), 1070 (w), 1152 (w), 1203 (m), 1228 (s), 1246 (m), 1267 (w), 1287 (m), 1361 (m), 1379 (w), 1391 (m), 1461 (m), 1476 (m), 1588 (m), 2866 (w), 2903 (w), 2953 (m).

II. NMR spectra

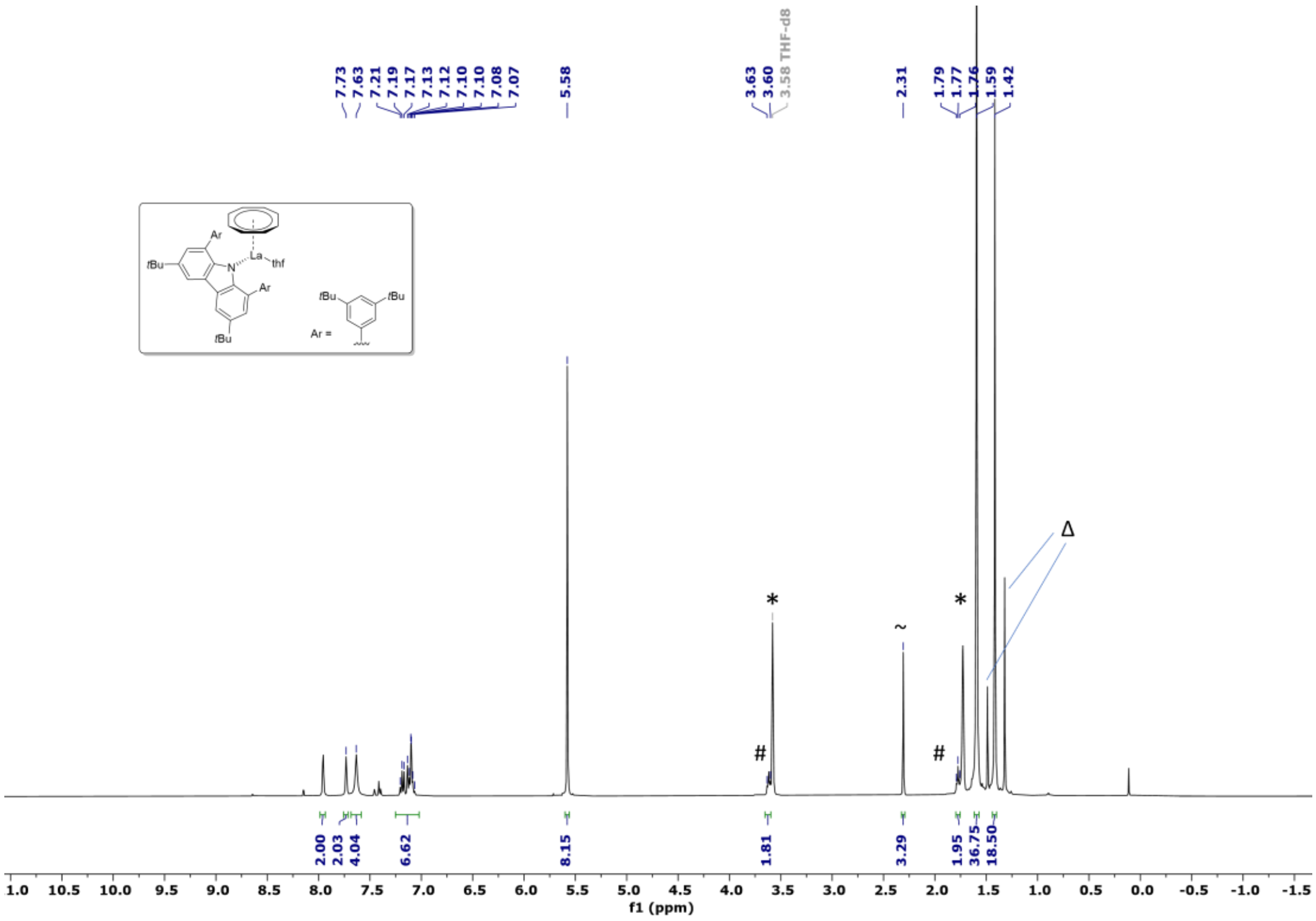

Figure S1. ^{1}H NMR spectrum of complex **2La** in THF-d_8.

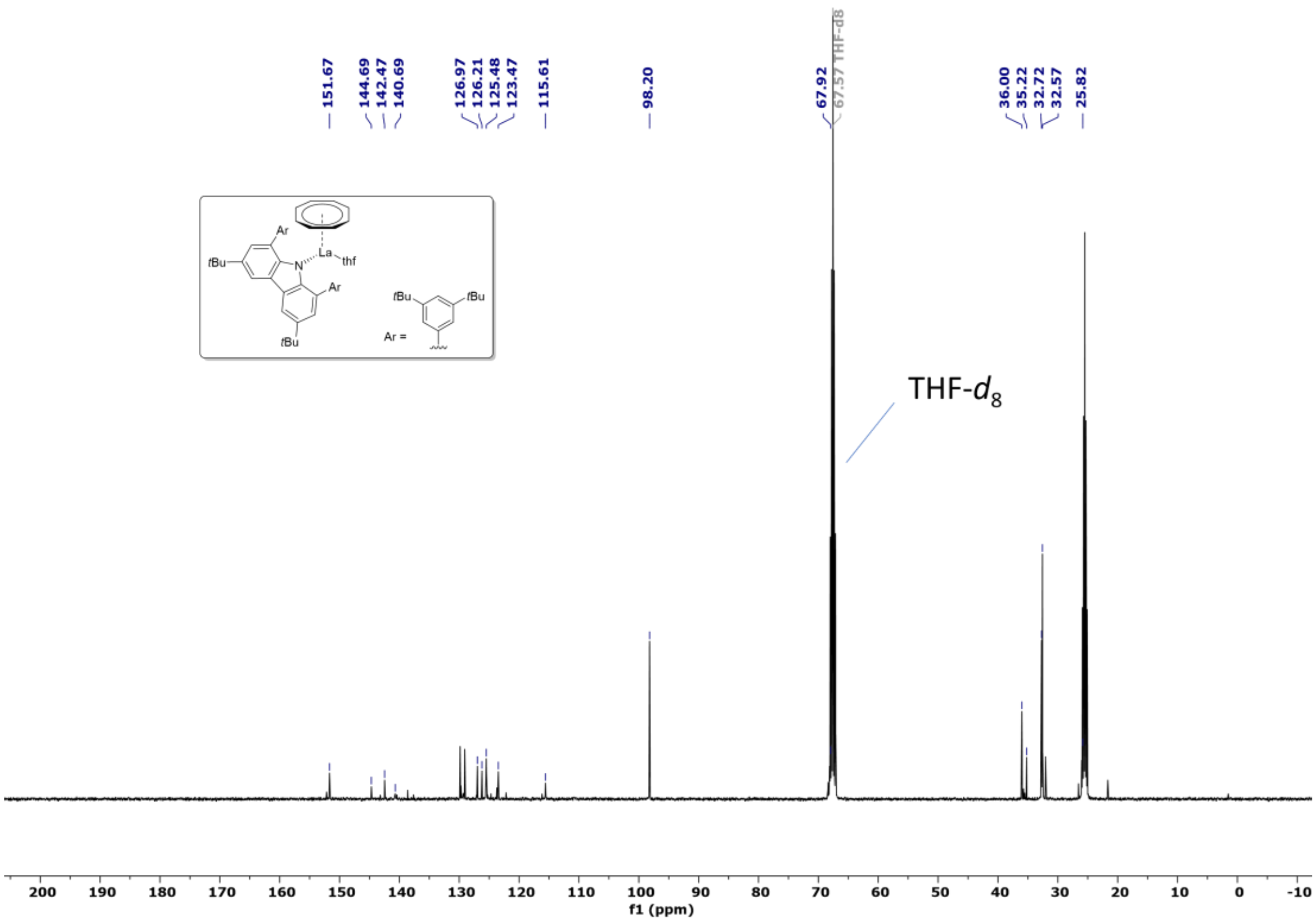

Figure S2. $^{13}C\{^{1}H\}$ NMR spectrum of complex **2La** in THF-d_8.

III. IR spectra

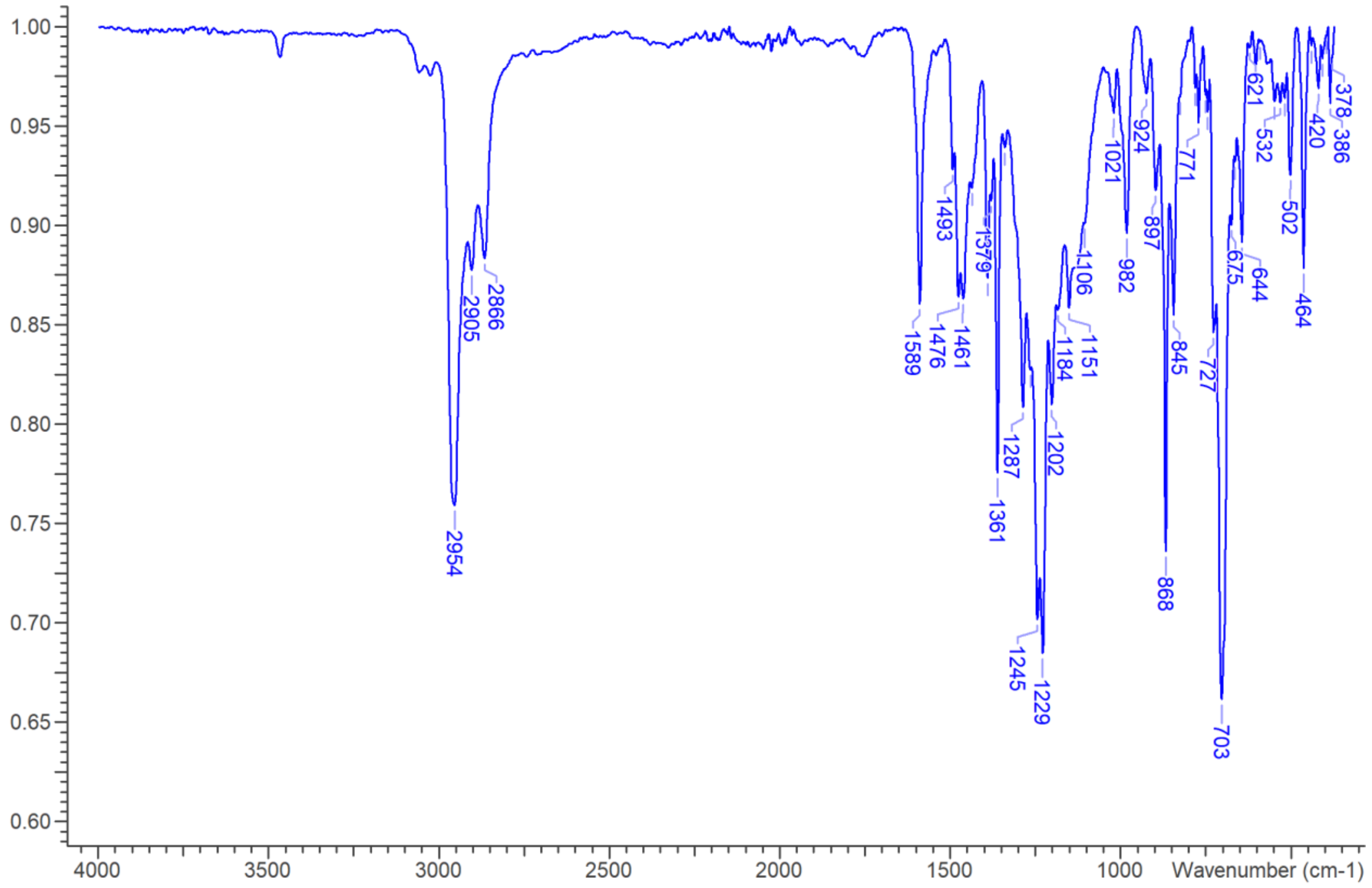

Figure S3. IR spectrum of complex **2La**.

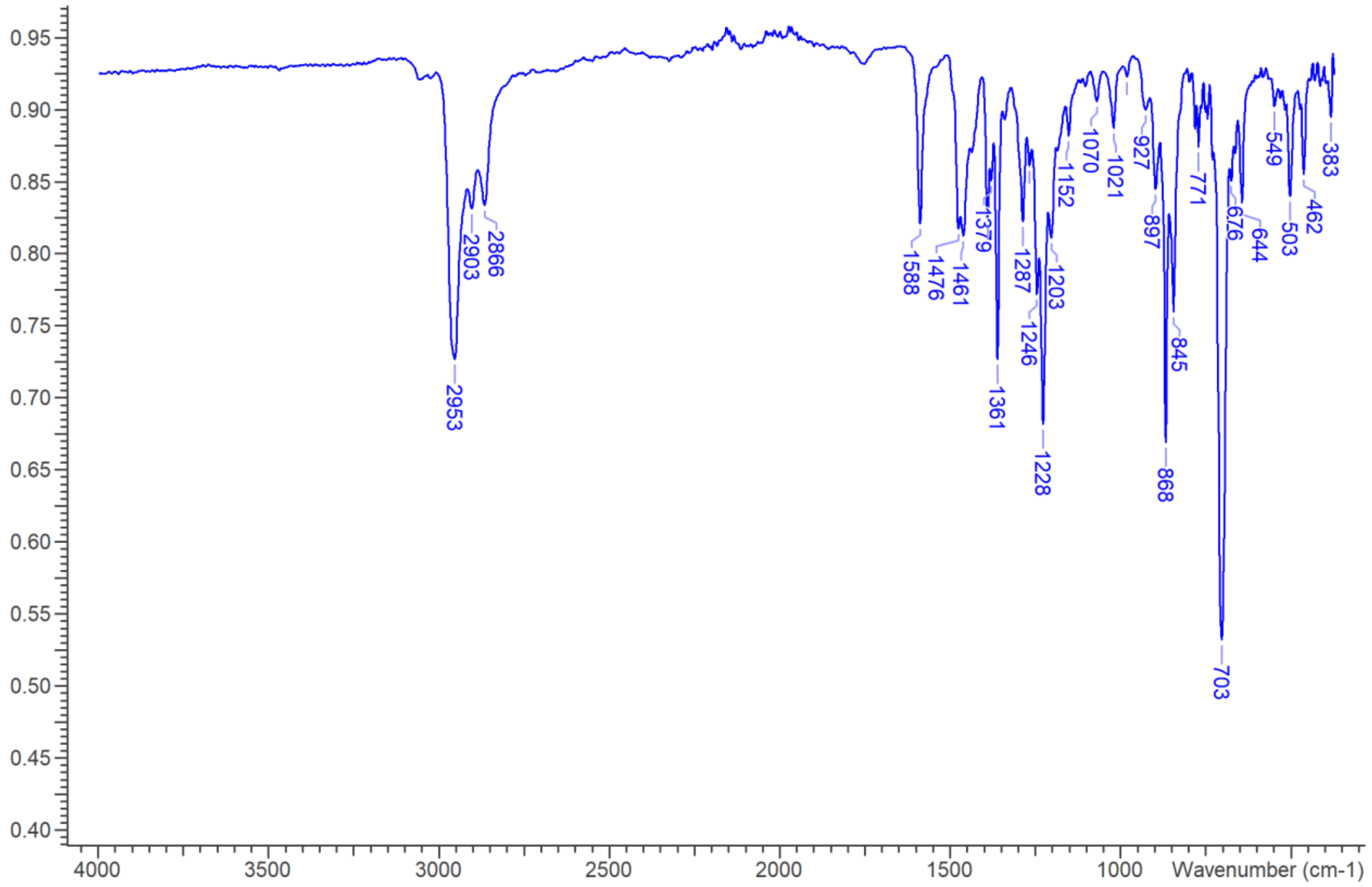

Figure S4. IR spectrum of complex **2Tb**.

IV. X-ray crystallography

IV.1 General methods

Suitable crystals for the X-ray analysis of all compounds were obtained as described above. A suitable crystal was covered in mineral oil (Aldrich) and mounted on a glass fibre. The crystal was transferred directly to the cold stream of a STOE StadiVari (100 K or 110 K) diffractometer. All structures were solved by using the program SHELXS/T[3,4] and Olex2.[5] The remaining non-hydrogen atoms were located from successive difference Fourier map calculations. The refinements were carried out by using full-matrix least-squares techniques on F_o^2 by using the program SHELXL.[3,4] The H-atoms were introduced into the geometrically calculated positions (SHELXL procedures) unless otherwise stated and refined riding on the corresponding parent atoms. In each case, the locations of the largest peaks in the final difference Fourier map calculations, as well as the magnitude of the residual electron densities, were of no chemical significance. Specific comments for each data set are given below. Summary of the crystal data, data collection and refinement for compounds are given in Table S1.

Crystallographic data for the structures reported in this paper have been deposited with the Cambridge Crystallographic Data Centre as a supplementary publication no. CCDC 2476612-2476615. Copies of the data can be obtained free of charge on application to CCDC, 12 Union Road, Cambridge CB21EZ, UK (fax: (+(44)1223-336-033; email: deposit@ccdc.cam.ac.uk).

The following special details were applied to the models of the structures:

In all disordered parts of the structures, anchor atoms were constrained with EADP, while all other ones were restrained with SIMU 0.01 0.02.

In the structure of **1La**, the whole molecule is disordered over two equally populated (0.5/0.5) and symmetry related positions with La–N being on the mirror plane. Additionally, two tBu groups C13–C16 and C17–C20 are disordered over two positions with occupancy ratio of 0.34/0.16 and 0.28/0.22, respectively.

In the structure of **1Tb**, four *t*Bu groups (C27–C30, C36–C39, C39–C42, C49–C52) is disordered over two positions with occupancy ratio of 0.79/0.21, 0.65/0.35, 0.59/0.41, 0.52/0.48, as well as one COT (0.62/0.38) and two toluene molecules (C61–C67 0.85/0.15, C68–C75 0.94/0.06).

In the structure of **2La**, carbazole moieties Cbz(N1) and Cbz(N2) were modelled as disordered (0.57/0.43, 0.53/0.47). Both COT rings are disordered as well (0.55/0.45, 0.51/0.49). Co-crystallised toluene molecules were removed with the SQUEEZE algorithm.

In the structure of **2Tb**, eight *t*Bu groups (C39–C42, C47–C50, C51–C54, C65–C68, C91–C94, C95–C98, C213–C216) are disordered over two positions with occupancy ratio of 0.69/0.31, 0.75/0.25, 0.90/0.10, 0.50/0.50, 0.53/0.47, 0.51/0.49, 0.59/0.41; One COT ring (C1–C8) is disordered over two positions with an occupancy ratio of 0.59/0.41; One toluene molecule (C117–C123) is disordered over three positions with an occupancy ratio of 0.41/0.39/0.18.

Table S1. Summary of crystal data.

**Compound**	**1La**	**1Tb**	**2La**	**2Tb**
CCDC #	2476612	2476613	2476614	2476615
Formula	$C_{68}LaO_3NH_{96}$	$C_{74}H_{96}NOTb$	$C_{137}H_{176}La_2N_2O$	$C_{123}H_{160}N_2OTb_2$
$D_{calc.}$/ g cm^{-3}	1.204	1.202	1.164	1.235
μ/mm^{-1}	0.739	5.970	0.738	1.353
Formula Weight	1114.36	1174.43	2144.61	2000.36
Colour	colourless	green	colourless	yellow
Shape	block	block	block	block
Size/mm^3	0.10×0.07×0.03	0.15×0.12×0.10	0.26×0.14×0.05	0.14×0.11×0.09
T/K	130(2)	180(2)	180(2)	100(2)
Crystal System	orthorhombic	monoclinic	orthorhombic	monoclinic
Space Group	*Pnma*	$P2_1/n$	$Pna2_1$	$P2_1/n$
a/Å	17.1198(6)	13.3571(5)	25.5420(5)	12.2282(2)
b/Å	25.6373(9)	15.5153(6)	25.9897(6)	30.4466(7)
c/Å	14.0045(4)	31.4992(10)	18.4285(5)	29.1059(6)
α/°	90	90	90	90
β/°	90	96.245(3)	90	97.001(1)
γ/°	90	90	90	90
V/Å^3	6146.7(4)	6489.1(4)	12233.4(5)	10755.5(4)
Z	4	4	4	4
Z'	0.5	1	1	1
Wavelength/Å	0.71073	1.34143	0.71073	0.71073
Radiation type	MoK_α	GaK_α	MoK_α	MoK_α
Θ_{min}/°	1.879	3.489	2.077	2.162
Θ_{max}/°	30.188	59.996	27.497	27.500
Measured Refl's.	35917	62733	90864	74719
Indep't Refl's	7862	14456	27850	24627
Refl's I≥2 σ(I)	5095	10284	13911	17966
R_{int}	0.0622	0.0476	0.0481	0.0252
Parameters	717	1053	2186	1627
Restraints	1337	1678	6455	2354
Largest Peak	0.722	0.701	0.807	1.622
Deepest Hole	-1.450	-0.519	-0.330	-2.528
GooF	1.040	1.034	0.919	1.018
wR_2 (all data)	0.1082	0.1051	0.1322	0.0900
wR_2	0.0907	0.0959	0.1077	0.0806
R_1 (all data)	0.1001	0.0735	0.1065	0.0648
R_1	0.0459	0.0440	0.0458	0.0400

IV.2 Summary of crystal data

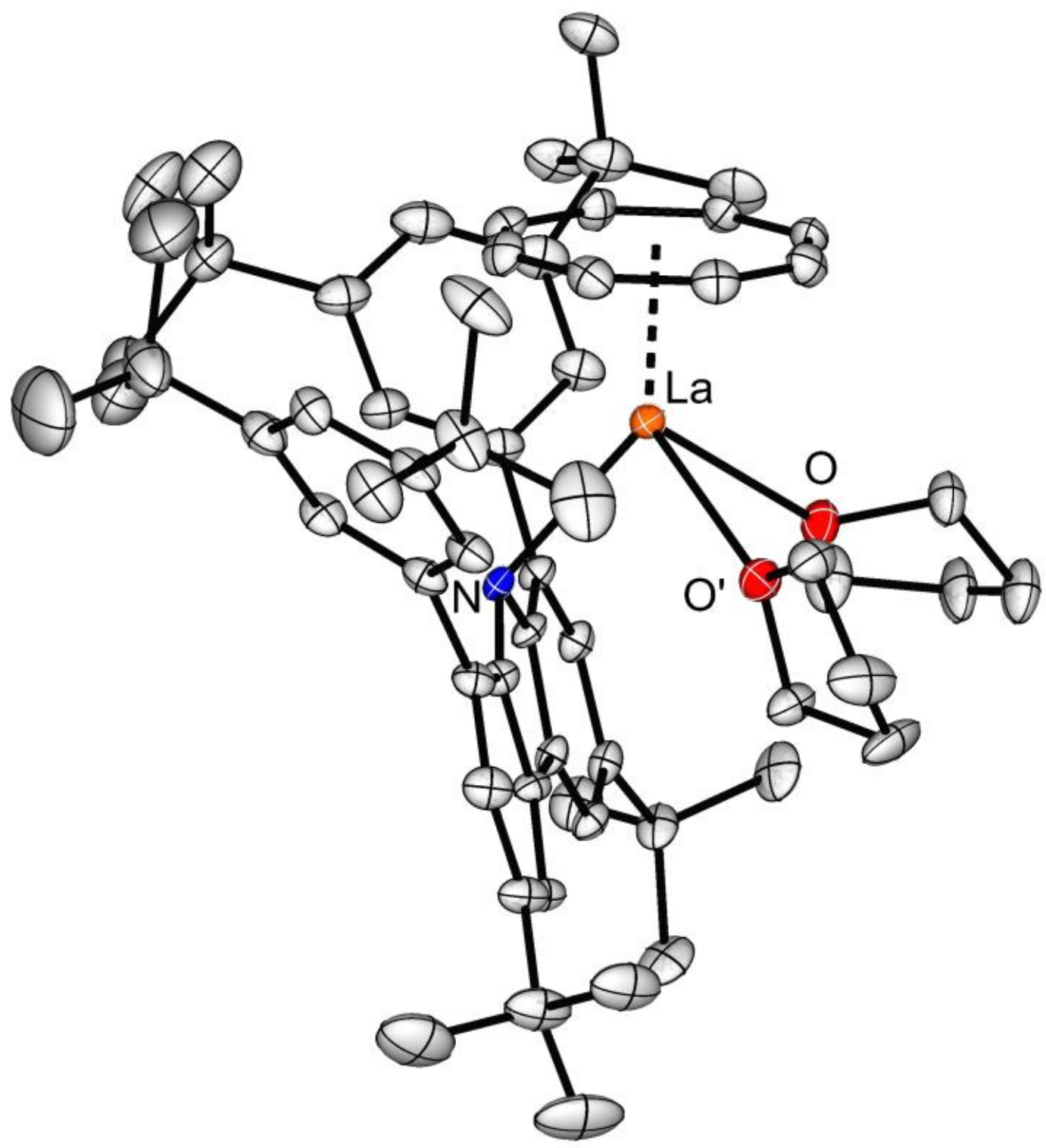

Figure 5. Molecular structure of the complex **1La** in the solid state with thermal ellipsoids at 40% level. All hydrogen atoms and the uncoordinated THF molecule are omitted for clarity. Selected bond distances [Å] and angles [°]: La–C_{COT} 2.026, La–O 2.627(2), La–N 2.579(3); O–La–O’ 69.54(11), O–La–N 90.39(8).

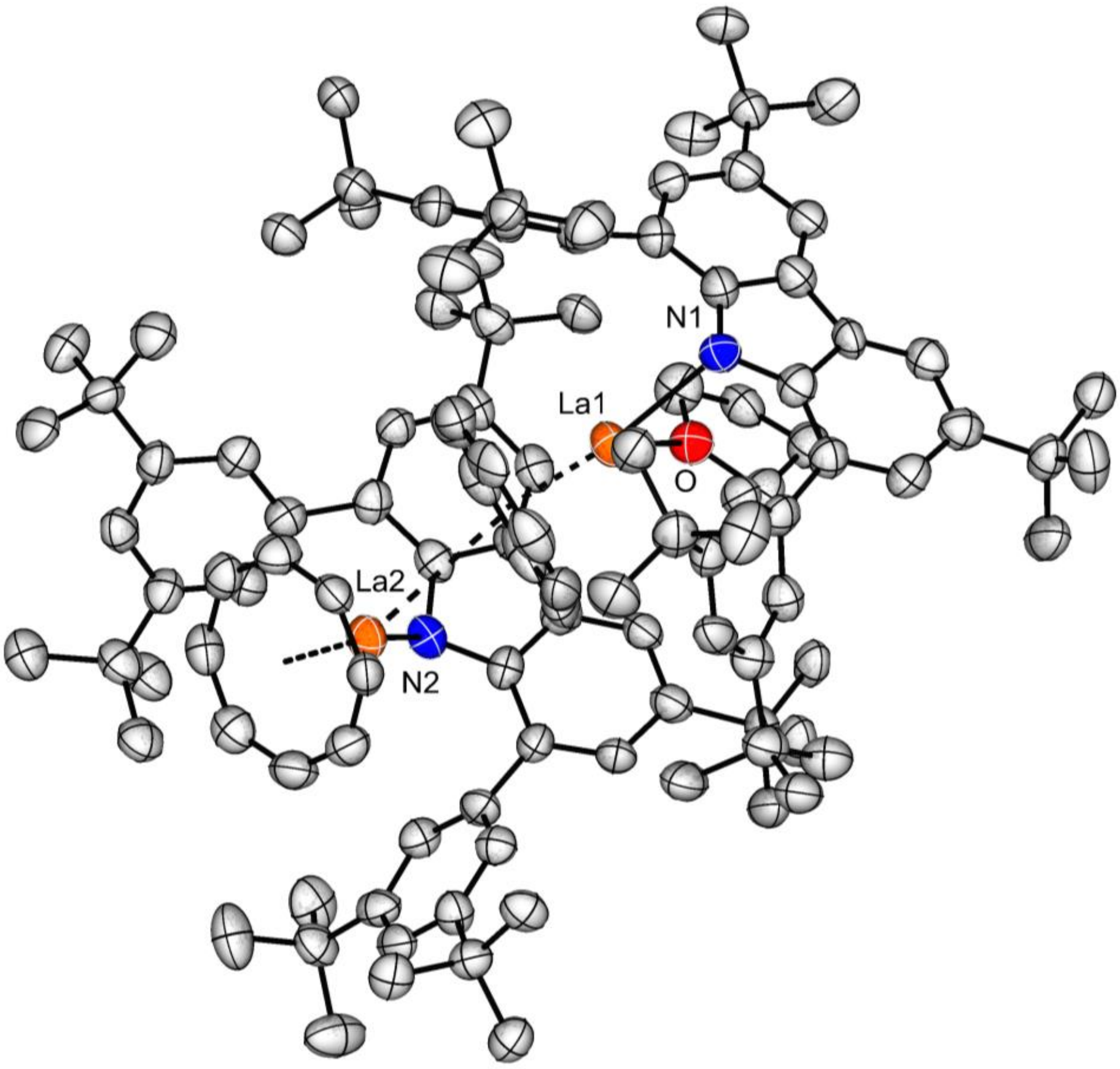

Figure S6. Molecular structure of the complex **2La** in the solid state with thermal ellipsoids at 40% level. All hydrogen atoms and the uncoordinated toluene molecules are omitted for clarity. Selected bond distances [Å] and angles [°]: La1–C_{COT} 2.009, La2–C_{COT} 1.973, La1–O 2.537(5), La1–N1 2.48(3), 2.49(3) O–La1–N1 78.2(6).

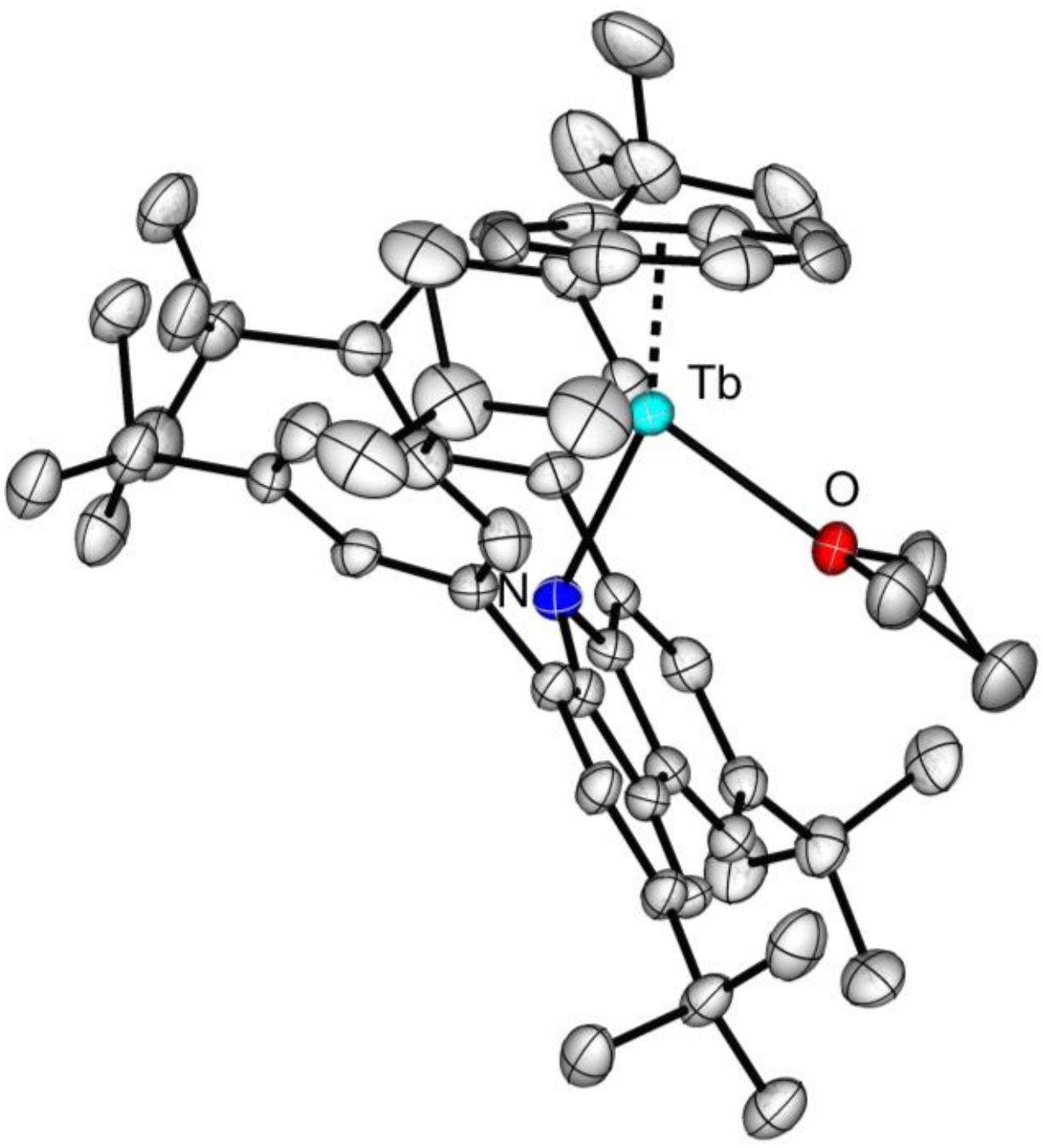

Figure S7. Molecular structure of the complex **1Tb** in the solid state with thermal ellipsoids at 40% level. All hydrogen atoms are omitted for clarity. Selected bond distances [Å] and angles [°]: Tb–C_{COT} 1.796, Tb–O 2.397(4), Tb–N 2.349(4); O–Tb–N 80.7(2).

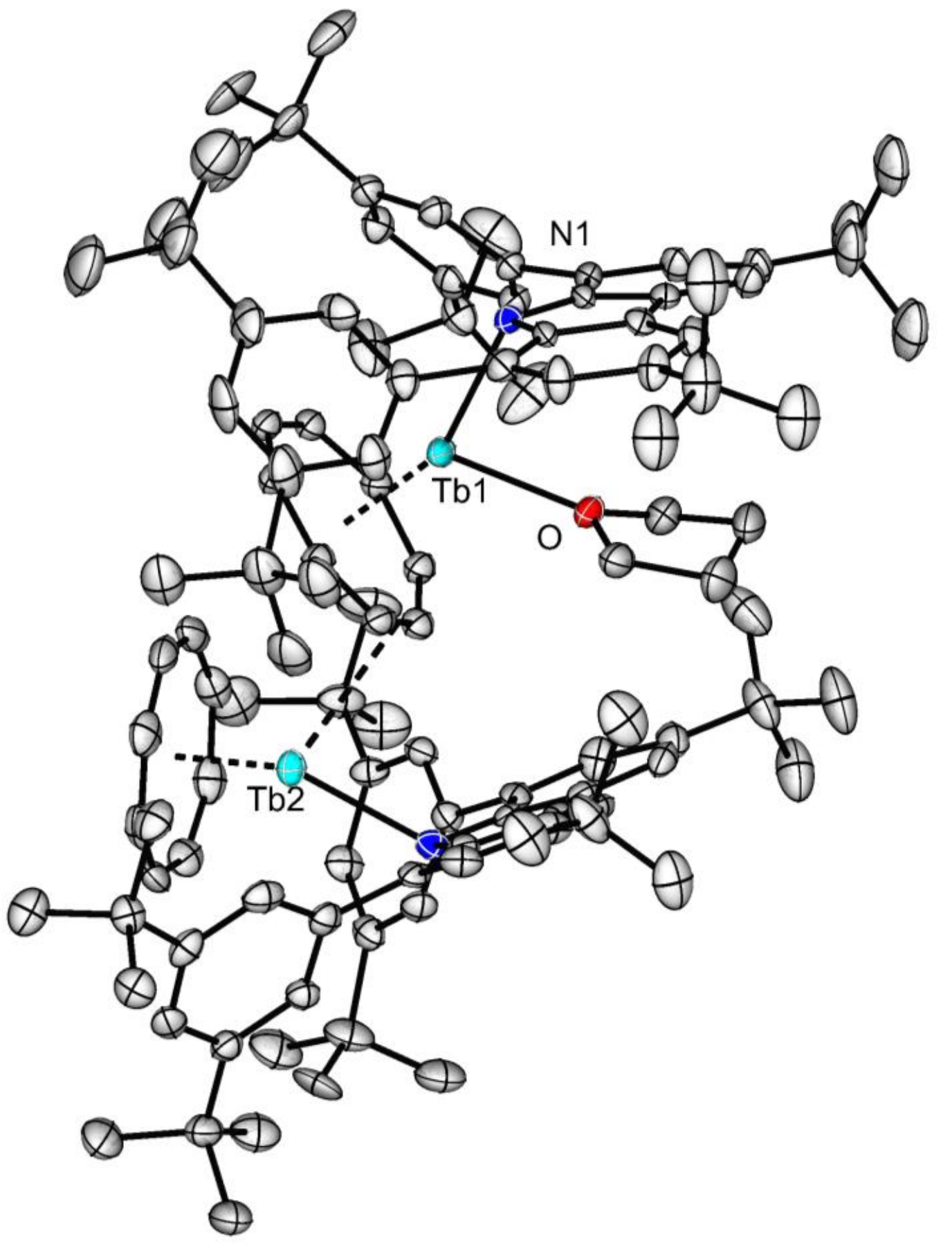

Figure S8. Molecular structure of the complex **2Tb** in the solid state with thermal ellipsoids at 40% level. All hydrogen atoms and the uncoordinated toluene molecule are omitted for clarity. Selected bond distances [Å] and angles [°]: Tb1–C_{COT} 1.820, Tb2–C_{COT} 1.773, Tb1–O 2.373(4), Tb1–N1 2.335(5), Tb2–N2 2.358(5); O–Tb1–N1 81.8(2).

V. Photoluminescence

V.1 General methods

Two devices were used for photoluminescence experiments:

1) PTI QuantaMaster^{TM} 8075-22 fluorometer with double monochromator (Horiba Jobin Yvon GmbH). Solid samples (crystalline powders) were measured as dispersions in a thin layer of viscous polyfluoroether oil (ABCR GmbH) placed between two 1 mm thin quartz plates. The latter were mounted on the holder and emission was collected at ca. 30° angle relative to the excitation light beam. For detection a R928 photomultiplier (Horiba Jobin Yvon GmbH) was used. All emission spectra were corrected for the wavelength dependent response of the spectrometer and detector. Emission decay traces were recorded with a Delta Diode^{TM} (HORIBA Jobin Yvon GmbH, Model DD-375L, λ_{Exc} = 375 nm, pulse width 70 ps, power 1.8 mW) or for lifetimes > 6 µs with a PTI XenonFlash^{TM} (modulation frequency 300 Hz). Several thousand traces were acquired and averaged.

2) Photoluminescence (PL) measurements were performed with Horiba Jobin Fluorolog-QM and Jobin Yvon Fluorolog-322 spectrometers. The first instrument included a liquid nitrogen optical Dewar cell for measurements at 77 K (of solid samples as well as of fast-frozen solutions). The second spectrometer included a closed-cycle optical cryostat (based on a Cryomech PT403 cryocooler) for temperature-dependent PL measurements down to T = 3 K. Solid (polycrystalline) probes of the air-sensitive compounds studied in this work were prepared for PL in an argon glovebox as dispersions in a thin layer of perfluoroether oil between two quartz plates, which were then quickly transferred into the cryostat and put under vacuum. The same sample preparation was applied to estimate a quantum efficiency of (weak) emission of **2Tb** at room temperature, using an integrating sphere placed into the sample chamber of Fluorolog-322. This measurement was done in air within ~5 min in order to minimize sample degradation. Solutions were transferred into sealable cuvettes or capillaries using Schlenk techniques under exclusion of air. All emission spectra were corrected for the wavelength-dependent response of the spectrometer and detector (in relative photon flux units). Emission decays were recorded either using the TCSPC technique and sub-ns pulsed laser diodes for excitation in the UV-blue spectral range (Fluorolog-QM) or with a fast digital oscilloscope connected to the detector (photomultiplier) and a ns-pulsed nitrogen laser for excitation at 337 nm (Fluorolog-322). For high-resolution emission spectroscopy, a Dilor XY 500 triple spectrograph equipped with a CCD camera was applied. It was set into the additive mode, providing a total focal length of 1.5 m and a spectral resolution of ca. 0.015 nm (0.5 cm^{-1} at 550 nm) with the 1800 mm^{-1} diffraction gratings. The spectrograph and a 375 nm laser diode for excitation were fiber-coupled to an optical head which was positioned to be focused on a sample in the cryostat.

V.2 PL spectra

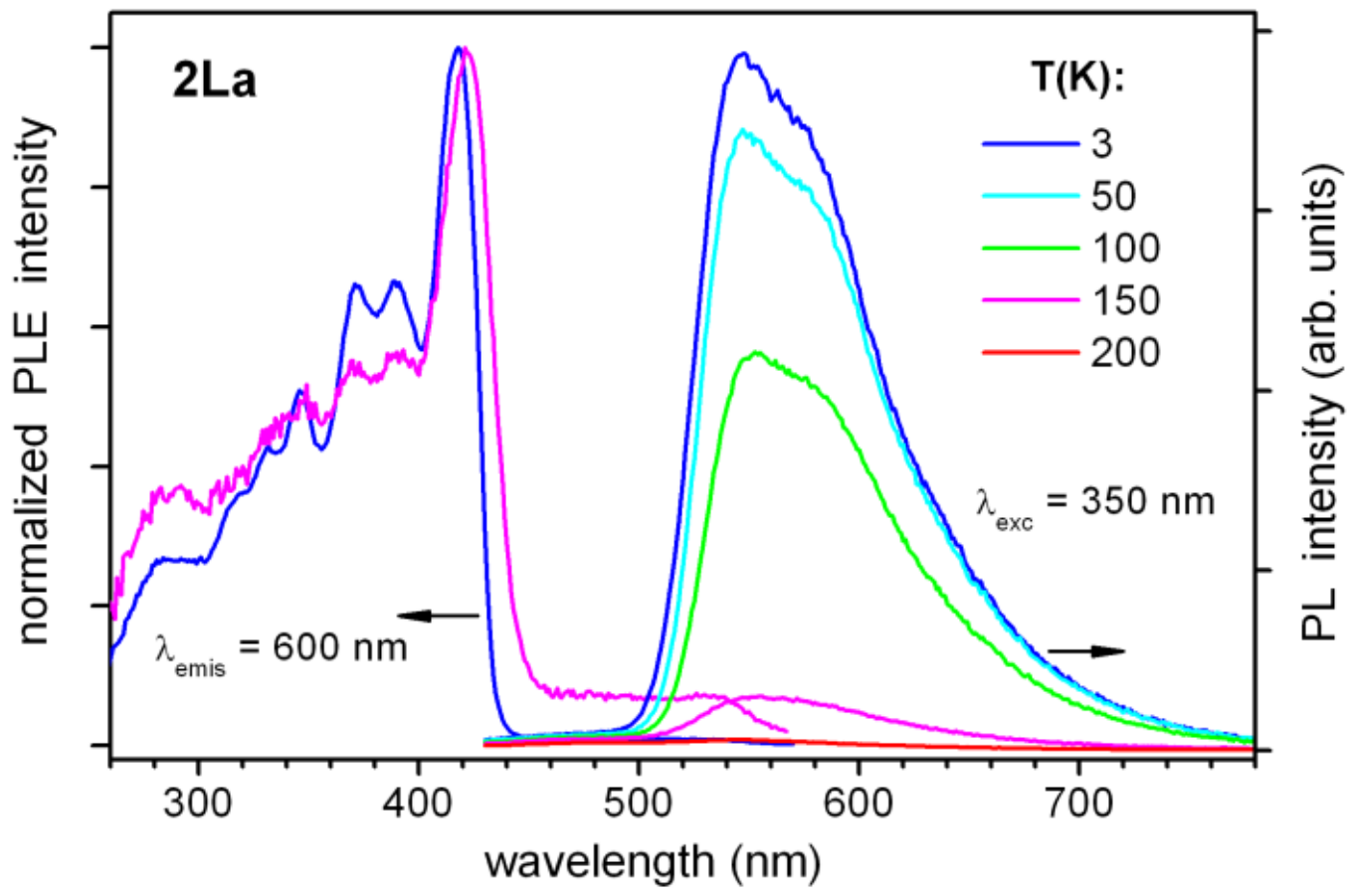

Figure *S*9. PL emission and excitation (PLE) spectra of solid (polycrystalline) **2La** at different temperatures between 3.0 and 200 K. The spectra were excited/recorded at 350/600 nm, respectively.

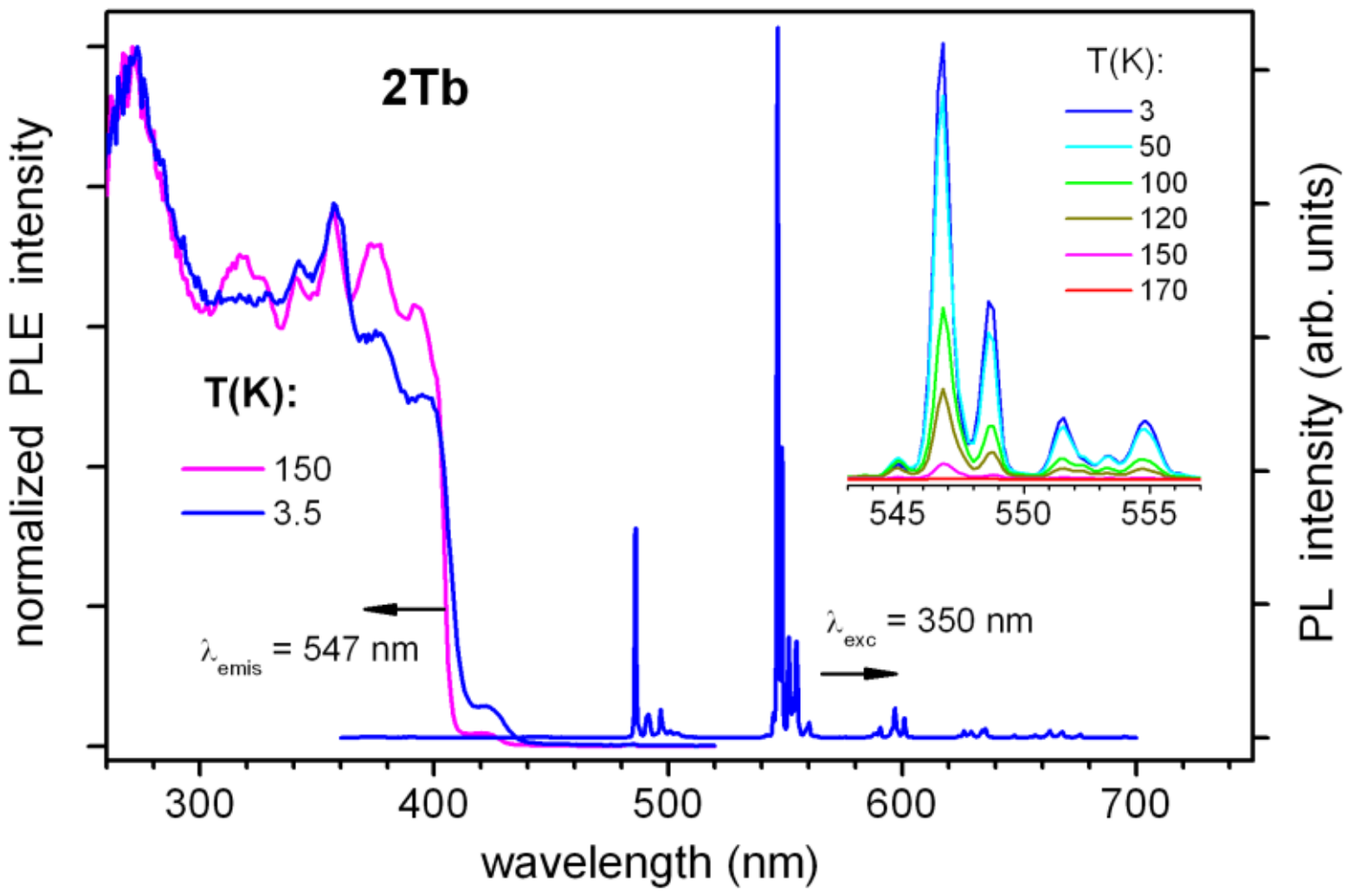

Figure *S*10. PL emission and excitation (PLE) spectra of solid (polycrystalline) **2Tb** at 3.5 and 150 K. The spectra were excited/ recorded at 350/ 547 nm, respectively. The insert shows a temperature dependence of the major emission band at ca. 547 nm between 3.5 K (relatively high intensity) and 170 K (strong decrease in intensity).

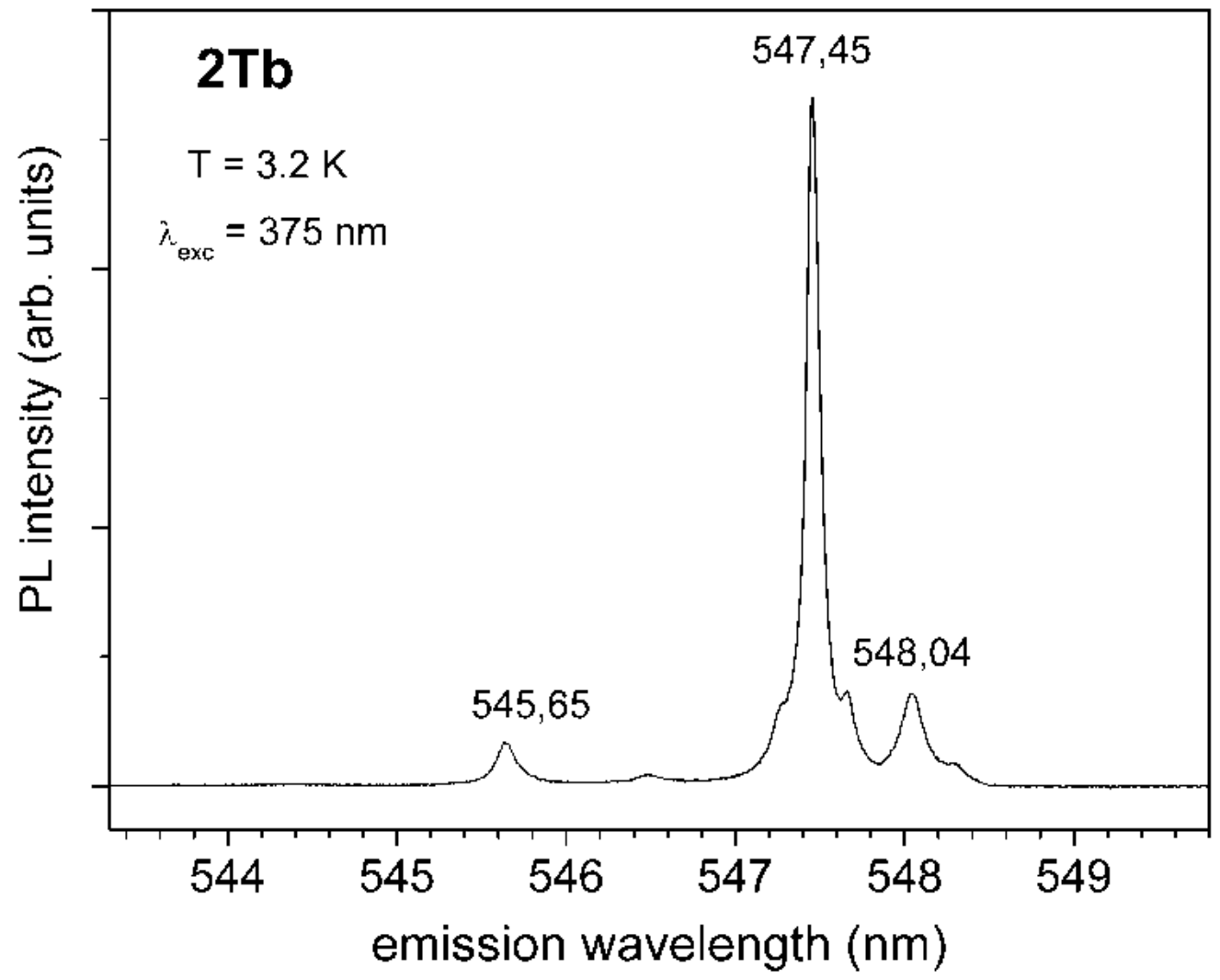

Figure *S*11. High-resolution emission spectrum of solid (polycrystalline) **2Tb** at T = 3.2 K, corresponding to the $^5D_4 \rightarrow {}^7F_5$ transition in the spectral range between 543 and 550 nm. A laser diode (~2 mW power) was used for excitation at 375 nm.

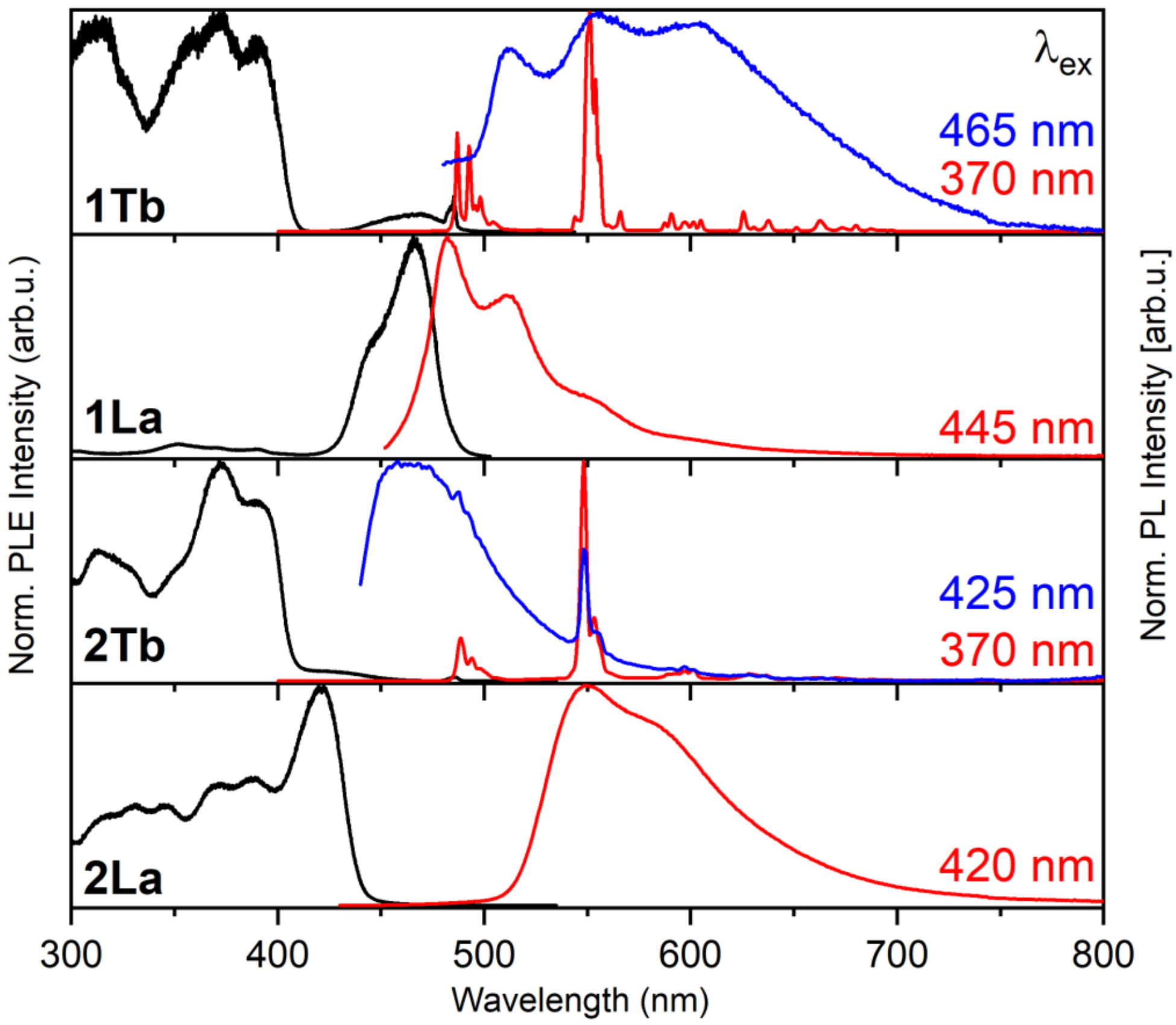

Figure *S*12. PLE and PL spectra of **1Tb** and **1La** (in frozen THF solution) and **2Tb** and **2La** (as crystalline solids) at 77 K. Black: PLE spectra; red: primary PL spectrum; blue: PL spectrum obtained with lower energy excitation.

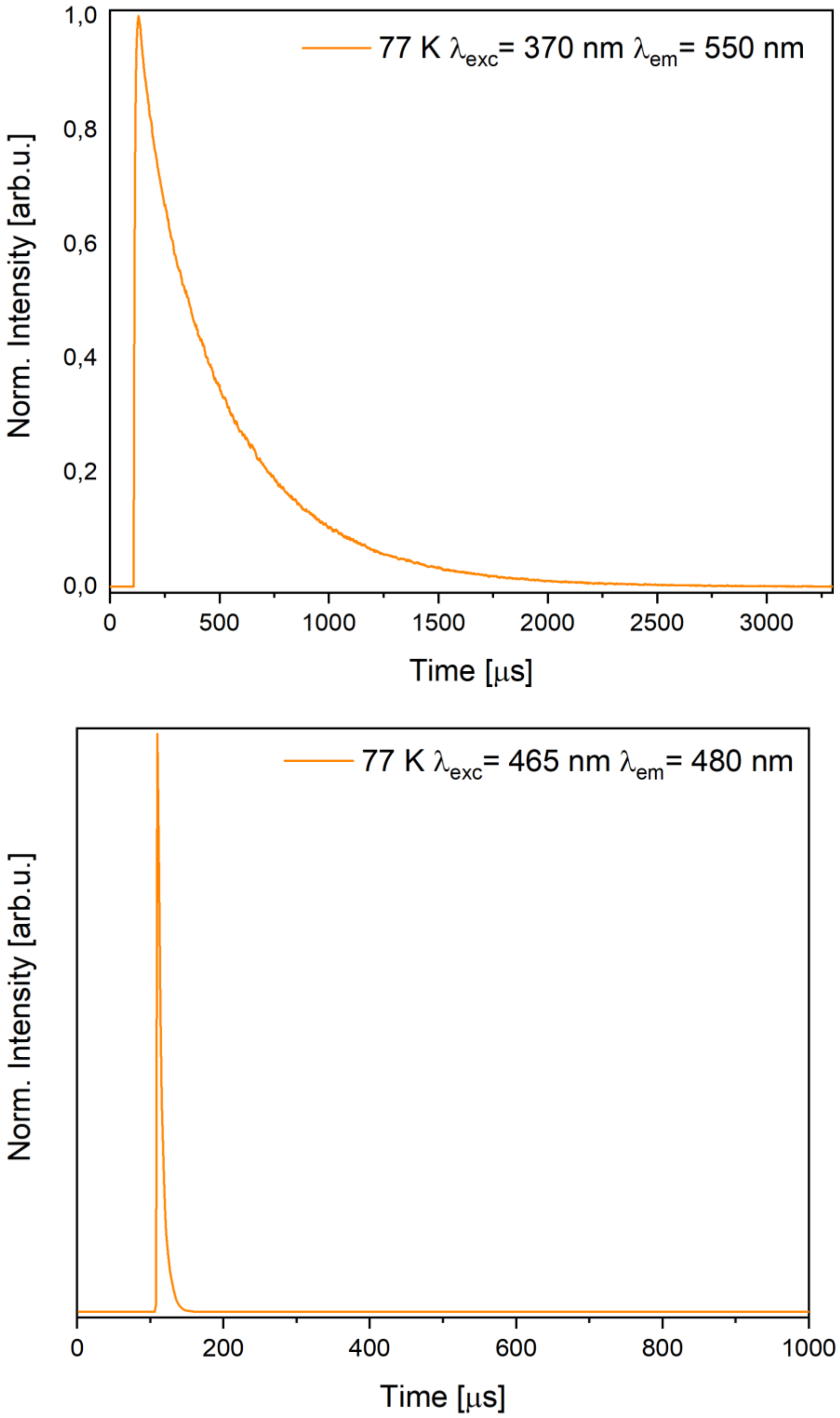

Figure *S*13. Emission decay of **1Tb** (top, t1 376 μs in single exponential fit) and **1La** (bottom, t1 6.06 μs in single exponential fit) in frozen THF solution.

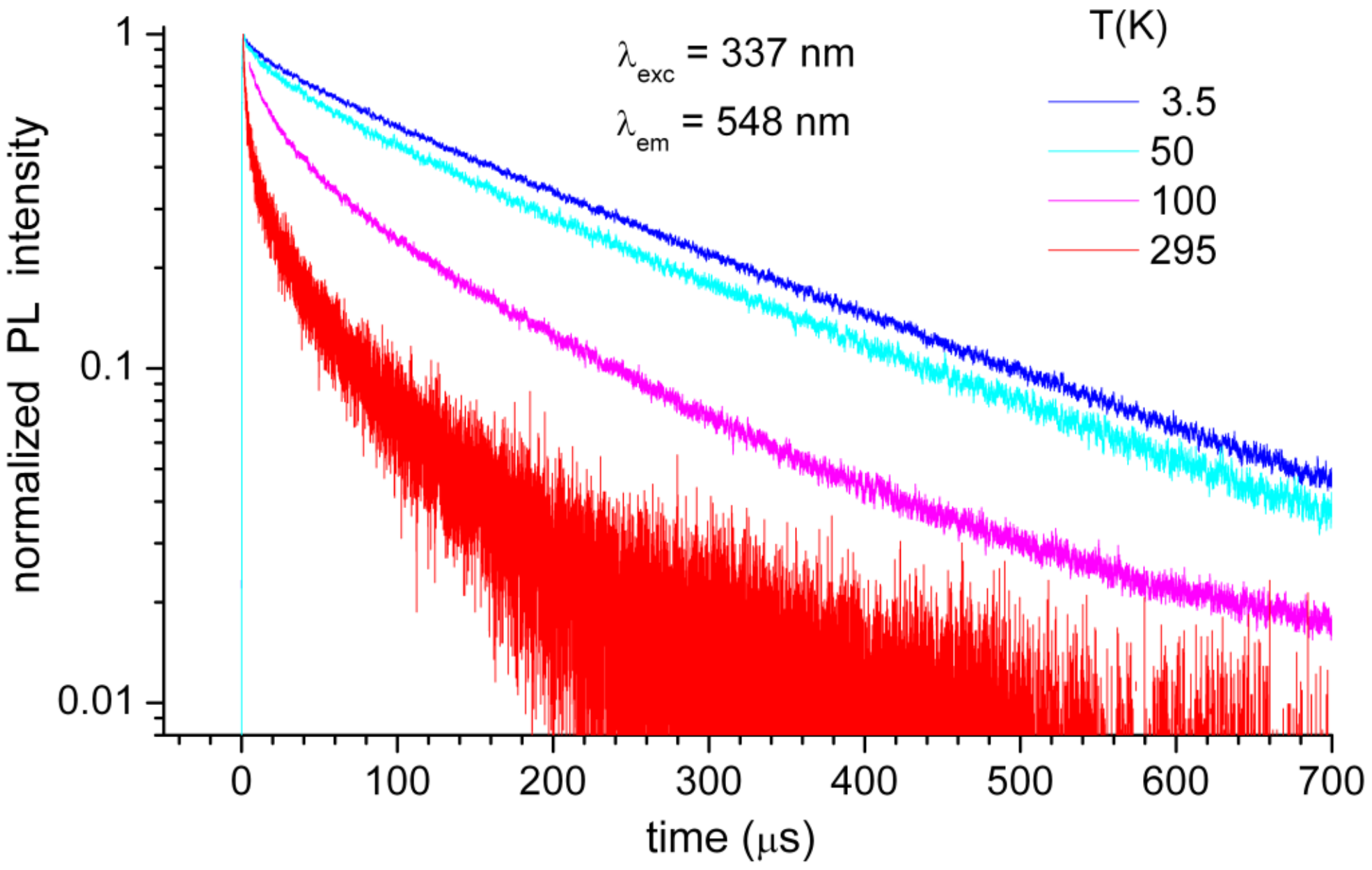

Figure *S*14. Decay traces of Tb(III) emission in solid **2Tb** at different temperatures, excited with a ns-pulsed laser at 337 nm and recorded at 548 nm. A low signal-to-noise ratio of the decay trace at 295 K (a very similar trace was also acquired at 200 K, not shown) is due to a strong decrease of the Tb(III) emission intensity above 100 K (see text). Biexponential fits of the decay traces yield the average lifetimes of 205, 180, 85, 45 and 48 μs at 3.5, 50, 100, 200 and 295 K, respectively.

VI. Quantum chemical calculations

VI.1 Computational Details

For **1La** and **1Tb**, time-dependent density functional theory (TDDFT) calculations were carried out with the program package TURBOMOLE V7.9[6] at the (one-electron) exact two-component decoupling level (X2C) in the diagonal local approximation for the U matrix (DLU),[7] at scalar-relativisic level (i.e. neglecting spin-orbit coupling) and employing the finite nucleus model.[8] The PBE0 functional[9] was used with fine grids (gridsize 5a,[10]) and x2c-TZVPall-2c basis sets.[11] Energies and oscillator strengths were calculated for the 40 energetically lowest excitations (singlet excitations in **1La,** general spin-conserving excitations in **1Tb** which shows 6 unpaired (f-)electrons in the ground state). For **1La**, the lowest two excitation bands result from 30 excitations which are reasonably well separated from the next ones. Similar is true also for **1Tb**, but here additionally six f-f transitions of low energies (2000 to 40000 nm) are found; their calculated spectrum is shown in Figure S14. Images of the orbitals involved in these transitions are shown in Figures S15 and S16, details of the transitions are listed in Tables S2 and S3. The images of the molecular orbitals and difference densities were done using the software Chemcraft.[12]

VI.2 Spectrum of f-f- transitions in 1Tb

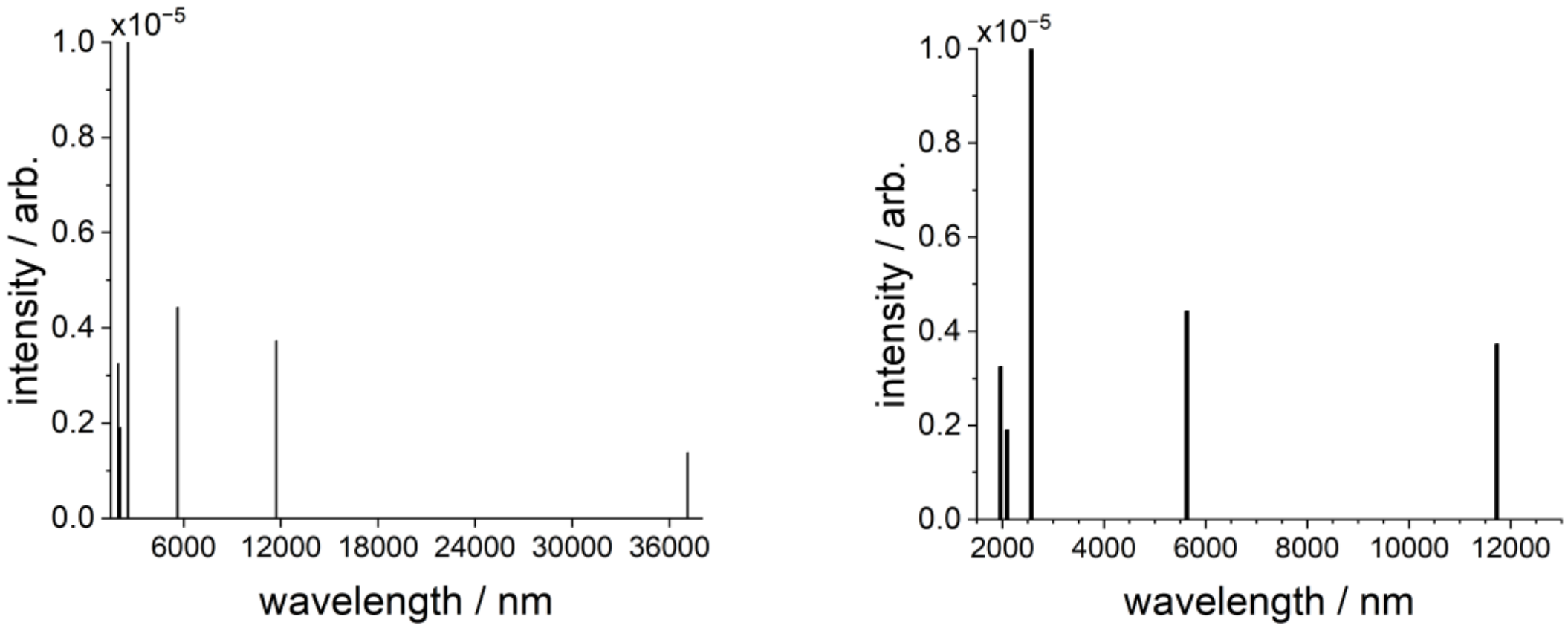

Figure *S*15. Section of the calculated excitation spectra of **1Tb** which shows very minor excitations in a range of roughly 37000 to 1960 nm (left: all six excitations; right: zoom-in on the five lower-lying excitations with the most dominant one at 2570 nm corresponding to a f–f transition with participation of HOMO-16 (see figure above).

VI.3 Molecular orbitals

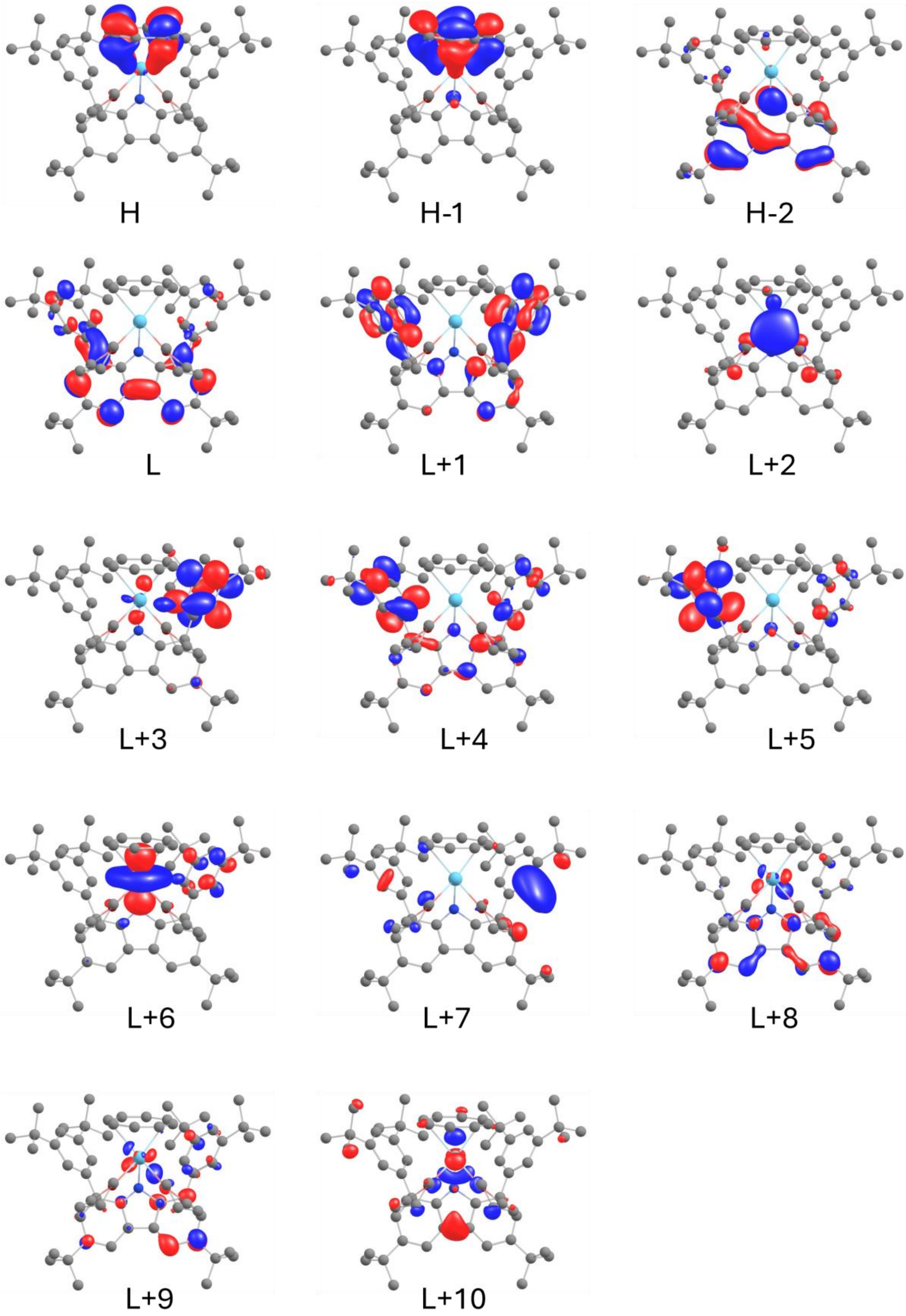

Figure *S*16. Frontier orbitals (HOMO-2 to LUMO+10) of **1La.** Contours are drawn at 0.04 a.u.

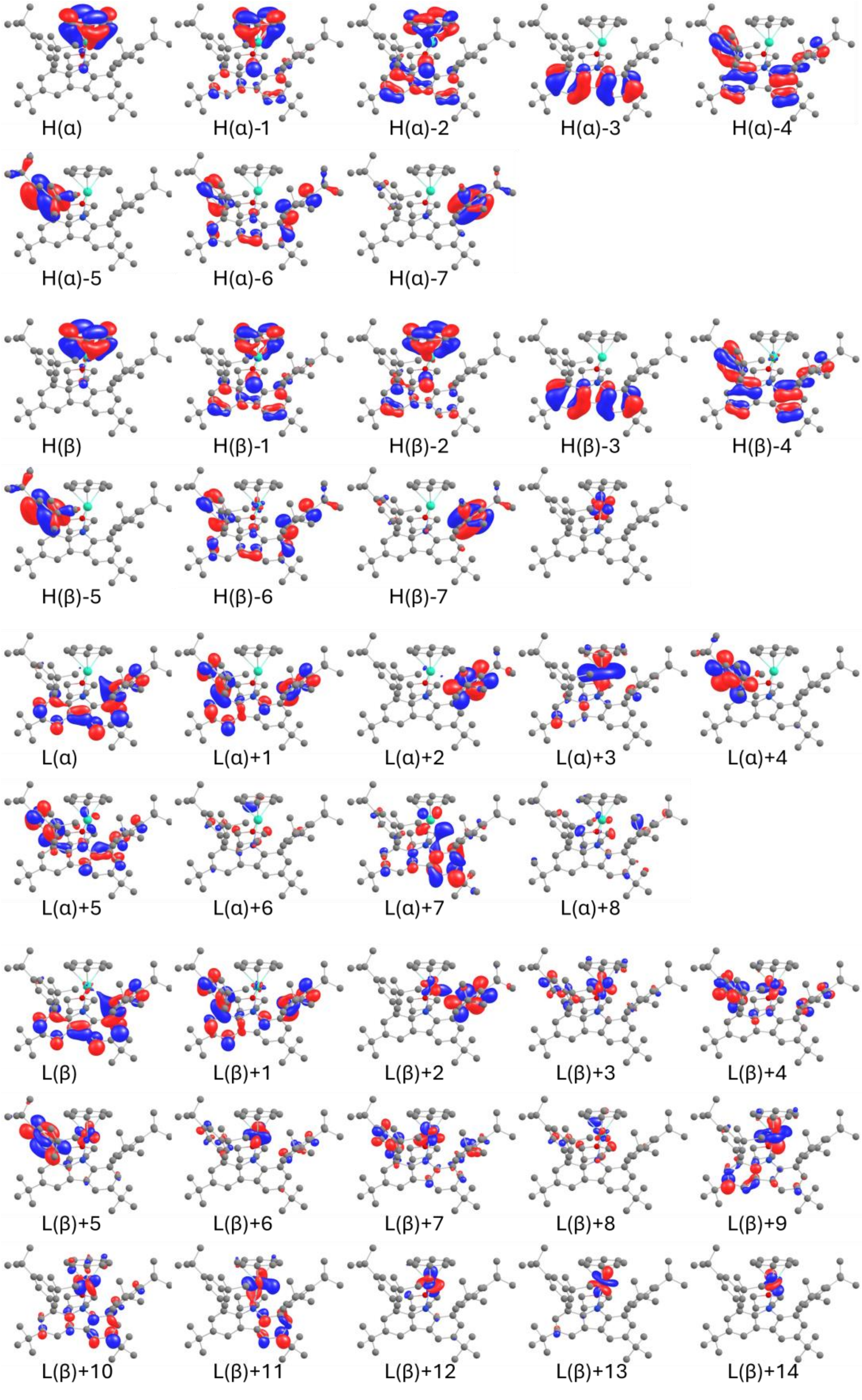

Figure *S*17. Frontier orbitals separately listed for majority spin (α) and minority spin (β) of **1Tb**. See also Figure Q1.

VI.4 Electronic transitions

Table S2. Energies, E, and oscillator strengths, O (velocity representation) for singlet excitations in **1La,** calculated at the PBE0/x2c-TZVPall-2c level of theory. In the subsequent columns the dominantly contributing orbital pairs, DCOP, (H: highest occupied orbital, L: lowest unoccupied orbital) and the size of their contribution, C, are listed. For images of the orbitals see Figure Q1.

	E/nm	O	DCOP	C / %
1	389.787	0.00223	H → L	96.1
2	381.701	0.10856	H-2 → L	69.9
			H-1 → L	27.0
3	373.213	0.01793	H-1 → L	69.3
			H-2 → L	26.7
4	339.773	0.00111	H → L+1	55.2
			H → L+6	23.5
			H → L+2	10.0
5	337.978	0.00060	H → L+1	43.4
			H → L+6	31.8
			H → L+2	11.9
6	330.432	0.04615	H-1 → L+1	51.6
			H-2 → L+1	40.6
7	327.853	0.00817	H-1 → L+6	38.4
			H-2 → L+1	20.9
			H-1 → L+2	15.5
			H-1 → L+3	14.0
8	326.396		H-1 → L+1	43.1
			H-2 → L+1	33.0
		0.00571	H-1 → L+6	12.5
9	312.662		H → L+2	68.0
		0.00008	H → L+3	19.8
10	309.402	0.10024	H-3 → L	71.2
11	303.552		H → L+3	52.6
		0.00061	H → L+6	24.7
12	302.852		H-1 → L+2	60.9
		0.00159	H-1 → L+3	13.8
13	301.644		H → L+4	59.3
		0.00350	H → L+5	36.1
14	299.090		H → L+5	58.7
		0.00319	H → L+4	35.2
15	296.066		H-2 → L+6	34.8
			H-2 → L+2	26.9
		0.02027	H-2 → L+3	19.7
16	293.954		H-1 → L+3	43.9
			H-1 → L+6	22.0
		0.00784	H-1 → L+4	15.0
17	292.779		H-1 → L+4	48.8
		0.01682	H-2 → L+4	23.0
18	291.185	0.00931	H-1 → L+5	75.1
19	289.493		H-2 → L+4	49.0
			H-1 → L+4	28.5
		0.01257	H-1 → L+5	11.3
20	287.290	0.01922	H-2 → L+5	81.6

21	285.866	0.04993	H-2 → L+3<br>H-2 → L+2	58.1<br>29.2
22	283.909	0.00706	H → L+7	82.9
23	281.105	0.04889	H-2 → L+6<br>H-2 → L+2	54.3<br>29.4
24	278.740	0.01664	H → L+8<br>H → L+9	43.8<br>25.9
25	277.981	0.00634	H-1 → L+7<br>H-1 → L+8<br>H-1 → L+9	50.6<br>21.8<br>14.7
26	273.706	0.07077	H-3 → L+1	67.9
27	273.059	0.00970	H-1 → L+7<br>H-1 → L+8<br>H-1 → L+9	36.6<br>26.8<br>11.8
28	272.385	0.00075	H → L+8<br>H → L+10<br>H → L+9	27.5<br>24.8<br>19.3
29	267.169	0.00385	H → L+10<br>H → L+9<br>H → L+8	37.3<br>32.8<br>12.5
30	267.088	0.19931	H-2 → L+8	61.9

Table S3. Excitations in **1Tb,** calculated at the PBE0/x2c-TZVPall-2c level of theory. See also Table S2. For images of the orbitals see Figure Q2.

	E / nm	O	DCOP	C / %
1	37129.256	0.0000014	H(β)-8 → L(β)+13<br>H(β)-8 → L(β)+10	42.6<br>23.3
2	11728.678	0.0000037	H(β)-8 → L(β)+13<br>H(β)-8 → L(β)+11<br>H(β)-8 → L(β)+10	33.4<br>15.2<br>11.8
3	5630.301	0.0000044	H(β)-8 → L(β)+14<br>H(β)-8 → L(β)+12	45.0<br>30.7
4	2569.626	0.0000240	H(β)-8 → L(β)+3<br>H(β)-8 → L(β)+2<br>H(β)-8 → L(β)+6	51.0<br>11.9<br>10.6
5	2095.861	0.0000019	H(β)-8 → L(β)+6<br>H(β)-8 → L(β)+5<br>H(β)-8 → L(β)+3<br>H(β)-8 → L(β)+7<br>H(β)-8 → L(β)+2	25.3<br>15.4<br>14.7<br>11.8<br>11.0
6	1961.976	0.0000033	H(β)-8 → L(β)+6<br>H(β)-8 → L(β)+7<br>H(β)-8 → L(β)+4	29.2<br>26.4<br>16.0
7	459.102	0.0000040	H(β)-1 → L(β)<br>H(α)-1 → L(α)<br>H(α)-2 → L(α)<br>H(β)-2 → L(β)	25.0<br>18.8<br>18.6<br>12.1

8	441.371	0.0000029	H(β)-3 → L(β)<br>H(α)-3 → L(α)	22.8<br>22.6
9	386.862	0.0008905	H(α) → L(α)	68.8
10	380.681	0.0065139	H(β) → L(β)<br>H(α) → L(α)	38.2<br>16.8
11	380.256	0.0053585	H(β) → L(β)<br>H(α)-1 → L(α)	34.9<br>9.7
12	370.9	0.0772636	H(α)-1 → L(α)<br>H(β)-1 → L(β)	37.3<br>37.3
13	367.218	0.0042947	H(α)-2 → L(α)<br>H(α)-1 → L(α)	39.4<br>14.2
14	363.942	0.0059333	H(β) → L(β)+3<br>H(β) → L(β)+2	22.9<br>11.0
15	362.958	0.0058360	H(β)-2 → L(β)<br>H(β) → L(β)+3<br>H(α)-2 → L(α)	42.6<br>9.6<br>9.0
16	360.491	0.0031255	H(β)-2 → L(β)<br>H(β) → L(β)+3	16.3<br>15.8
17	353.267	0.0005066	H(β) → L(β)+6<br>H(β) → L(β)+2<br>H(β) → L(β)+5<br>H(β) → L(β)+3	20.4<br>18.1<br>15.9<br>10.4
18	346.414	0.0005363	H(β)-1 → L(β)+3<br>H(β)-2 → L(β)+3	21.1<br>19.2
19	341.803	0.0000820	H(β)-2 → L(β)+1<br>H(α)-2 → L(α)+1	5.1<br>5.1
20	340.503	0.0006223	H(β) → L(β)+4<br>H(β) → L(β)+7<br>H(β) → L(β)+6	18.3<br>15.2<br>14.9
21	336.62	0.0009065	H(α) → L(α)+3	11.9
22	335.367	0.0002106	H(α) → L(α)+3	56.5
23	326.599	0.0013238	H(β)-1 → L(β)+6<br>H(β)-2 → L(β)+6	12.3<br>10.0
24	323.794	0.0121726	H(α) → L(α)+1	72.5
25	323.063	0.0089207	H(α)-1 → L(α)+3<br>H(α)-2 → L(α)+3	38.1<br>19.3
26	322.755	0.0070275	H(β) → L(β)+1	23.8
27	321.094	0.0008057	H(β) → L(β)+10	9.2
28	320.637	0.0131247	H(β) → L(β)+1<br>H(β) → L(β)+10	32.2<br>11.5
29	317.81	0.0169388	H(β)-1 → L(β)+1<br>H(α)-1 → L(α)+1<br>H(α)-2 → L(α)+1	24.3<br>22.4<br>10.9
30	312.538	0.0001608	H(α)-1 → L(α)+1	10.5
31	312.235	0.00098087	H(β)-3 → L(β)+1<br>H(α)-3 → L(α)+1	10.6<br>10.1
32	311.014	0.00104021	H(α)-2 → L(α)+1<br>H(α)-1 → L(α)+1	34.0<br>28.0
33	310.036	0.00939088	H(α) → L(α)+2	28.8
34	309.676	0.15602006	H(β)-3 → L(β)<br>H(α)-3 → L(α)	36.2<br>32.7

35	308.458	0.01338827	H(β)-2 → L(β)+1<br>H(β)-1 → L(β)+1	38.8<br>11.5
36	307.046	0.01055497	H(β)-1 → L(β)+2<br>H(β)-2 → L(β)+2<br>H(β)-2 → L(β)+1	16.4<br>12.9<br>10.1
37	303.954	0.00296392	H(β) → L(β)+13<br>H(β) → L(β)+9	47.9<br>19.8
38	301.823	0.00710899	H(β) → L(β)+14<br>H(β) → L(β)+12<br>H(α) → L(α)+2	17.0<br>14.4<br>10.5
39	300.697	0.00246693	H(β) → L(β)+14<br>H(β) → L(β)+12	22.2<br>17.2
40	299.669	0.01896481	H(α)-1 → L(α)+2	42.3

VII. References

[1] A. Hinz, *Chem. Eur. J.* **2019**, *25*, 3267-3271.

[2] a) K. Mashima, Y. Nakayama, A. Nakamura, N. Kanehisa, Y. Kai and H. Takaya, J. Organomet. Chem. **1994**, *473*, 85-91. b) L. Münzfeld, C. Schoo, S. Bestgen, E. Moreno-Pineda, R. Köppe, Nat. Commun. **2019**, *10*, 1-7.

[3] G. Sheldrick, *Acta Cryst. A* **2008**, *64*, 112-122.

[4] G. Sheldrick, *Acta Cryst. C* **2015**, *A71*, 3-8.

[5] O. V; Dolomanov, L, J, Bourhis, R. J. Gildea, J. A. K. Howard, H. Puschmann, *J. Appl. Crystallogr*. **2009**, *42*, 339-341.

[6] TURBOMOLE, Version 7.9 2024; a development of University of Karlsruhe and Forschungszentrum Karlsruhe GmbH 1989-2007, TURBOMOLE GmbH since 2007, available via www.turbomole.org, **2024**.

[7] D. Peng, N. Middendorf, F. Weigend, M. Reiher, *J. Chem. Phys*. **2013**, *138*, 184105.

[8] L. Visscher, K. Dyall, *At. Data Nucl. Data Tables*, **1997**, *67,* 207-224.

[9] J. P. Perdew, M. Ernzerhof, K. Burke, *J. Chem. Phys*. **1996**, *105*, 9982-9985

[10] Y.J. Franzke, R. Treß, T. M. Pazdera, F. Weigend, *Phys. Chem. Chem. Phys.* **2019**, *21*, 16658 - 16664.

[11] P. Pollak, F. Weigend, *J. Chem. Theory Comput*. **2017**, *13*, 3696-3705.

[12] Chemcraft - graphical software for visualization of quantum chemistry computations. Version 1.8, build 682, https://www.chemcraftprog.com.
